# *Clostridium sordellii* outer spore proteins maintain spore structural integrity and promote bacterial clearance from the gastrointestinal tract

**DOI:** 10.1371/journal.ppat.1007004

**Published:** 2018-04-18

**Authors:** Rebecca Rabi, Sarah Larcombe, Rommel Mathias, Sheena McGowan, Milena Awad, Dena Lyras

**Affiliations:** 1 Infection and Immunity Program, Monash Biomedicine Discovery Institute and Department of Microbiology, Monash University, Clayton, Victoria, Australia; 2 Infection and Immunity Program, Monash Biomedicine Discovery Institute and Department of Biochemistry and Molecular Biology, Monash University, Clayton, Victoria, Australia; Tufts University, UNITED STATES

## Abstract

Bacterial spores play an important role in disease initiation, transmission and persistence. In some species, the exosporium forms the outermost structure of the spore and provides the first point of contact between the spore and the environment. The exosporium may also be involved in spore adherence, protection and germination. *Clostridium sordellii* is a highly lethal, spore forming pathogen that causes soft-tissue infections, enteritis and toxic-shock syndrome. Despite the importance of *C*. *sordellii* spores in disease, spore proteins from this bacterium have not been defined or interrogated functionally. In this study, we identified the *C*. *sordellii* outer spore proteome and two of the identified proteins, CsA and CsB, were characterised using a genetic and phenotypic approach. Both proteins were essential for the correct formation and positioning of the *C*. *sordellii* spore coat and exosporium. The absence of CsA reduced sporulation levels and increased spore sensitivity to heat, sodium hydroxide and hydrochloric acid. By comparison, CsB was required for normal levels of spore adherence to cervical, but not vaginal, cells, with *csB* mutant spores having increased adherence properties. The establishment of a mouse infection model of the gastrointestinal tract for *C*. *sordellii* allowed the role of CsA and CsB to be interrogated in an infected host. Following the oral administration of spores to mice, the wild-type strain efficiently colonized the gastrointestinal tract, with the peak of bacterial numbers occurring at one day post-infection. Colonization was reduced by two logs at four days post-infection. By comparison, mice infected with the *csB* mutant did not show a reduction in bacterial numbers. We conclude that *C*. *sordellii* outer spore proteins are important for the structural and functional integrity of spores. Furthermore, outer spore proteins are required for wild-type levels of colonization during infection, possibly as a result of the role that the proteins play in spore structure and morphology.

## Author summary

*Clostridium sordellii* is a lethal pathogen in humans and animals and its spores are critical for initiating infection. Outer spore proteins in *Bacillus* and *Clostridium* species are important for spore fitness and pathogenesis, however, equivalent proteins in *C*. *sordellii* have not been identified and their role is unknown. In this study, we characterized two *C*. *sordellii* outer spore proteins, CsA and CsB, and showed that these proteins are required for correct spore structure and function. We also established the first mouse model of *C*. *sordellii* gastrointestinal tract infection. This model is physiologically relevant as *C*. *sordellii* can cause enteric disease in animals. Our model is unique in comparison to previous *C*. *sordellii* disease models in that spores, and not vegetative cells, are administered to reflect what likely occurs in naturally occurring infections. Using our model we showed that the absence of CsB allows a *csB* mutant strain to persist within the host. We also demonstrated that although *C*. *sordellii* CsA shares homology with the *Clostridium difficile* spore protein CdeC, these proteins contribute in different ways to spore phenotypes in the two bacterial hosts, highlighting the necessity of studying spore proteins in the cognate species from which they originate.

## Introduction

*Bacillus* and *Clostridium* bacterial species produce spores as a survival mechanism, in response to adverse conditions such as nutrient starvation [1, 2]. Spores are resistant in environmentally unfavourable conditions and allow bacteria to persist in conditions that do not allow the survival of metabolically active vegetative cells. In both *Bacillus* and *Clostridium* species, spores are critical for disease as they are often responsible for disease initiation, transmission and relapse [1–7].

*Clostridium sordellii* is a spore-forming bacterium that is responsible for severe human and animal diseases, including enteritis, bacteraemia and soft-tissue infections [8–12]. *C*. *sordellii* pathogenesis is toxin-mediated with two major toxins, TcsL and TcsH, responsible for host cell cytoskeletal disorganisation and death [12, 13]. Mutagenesis studies using a TcsL-producing strain have shown that TcsL is essential for *C*. *sordellii* pathogenesis since a *tcsL* mutant was avirulent in an animal infection model while the TcsL-producing parent strain resulted in severe disease and death [14]. *C*. *sordellii* spores are important in disease because they are the infectious particle that can initiate infections. Soft tissue infections in injecting-drug users, for example, are likely to be initiated by *C*. *sordellii* spores which contaminate black-tar heroin. Speed-balling and skin-popping performed by these drug users can lead to ischemia and necrosis, which are favourable for spore germination and outgrowth [15, 16]. Spores are also important in initiating diseases that result in post-partum and post-abortive clostridial toxic-shock. The uterus post-pregnancy contains amino acids, progesterone, and an elevated pH, which either trigger or enhance *C*. *sordellii* spore germination, resulting in spore outgrowth in the female reproductive tract and infection [17]. *C*. *sordellii*-mediated enteric diseases in animals are also likely to result from the ingestion of environmental spores that contaminate food or drinking water [9, 10].

The *C*. *sordellii* spore is a complex structure, made up of an inner spore surrounded by a baggy, balloon-like exosporium [18]. The area between the inner spore and exosporium is known as the interspace in *C*. *sordellii* and in some *Bacillus* species, such as *B*. *anthracis* [18, 19]. In *C*. *sordellii*, the inner spore is multilayered and composed of a core, inner membrane, germ cell wall, cortex and coat. The coat itself consists of three layers: the undercoat (also termed the basement layer [20, 21]), inner coat and outer coat [18]. The role of the inner spore proteins and structures in *C*. *sordellii* has not been studied, but in *Bacillus subtilis* and *Clostridioides* (previously *Clostridium* [22]) *difficile* they play a role in spore protection and germination [1, 2, 20]. The exosporium surrounding the inner spore is thought to be important as it is the first contact point between the spore and the environment [18, 23], however, very little is known about the functional role of this structure. In some species, the exosporium may play a role in spore protection against antibodies, degradative enzymes and host macrophages, and a role in regulating spore adherence to biotic surfaces [24–28]. Specific exosporial proteins may be responsible for some of these roles. For example, in *Bacillus anthracis*, the exosporial proteins elongation factor-Tu, enolase and arginase are thought to prevent spore opsonization or to provide protection against free radicals within macrophages, thus contributing to the survival of the spore or the germinating bacterium [24, 25]. Despite the importance of exosporial proteins, the *C*. *sordellii* spore proteome has not previously been characterised. Exosporial proteins have been identified in the closely related organism *C*. *difficile*, however, the functional role of most of these proteins is not known [29]. Of the few *C*. *difficile* proteins that have been characterised, CdeC was found to be essential for the proper assembly of the exosporium and coat layers [30]. Characterisation of spore proteins is required to better understand their functional and structural roles, and these studies are particularly important for exosporial and other outer spore proteins, since they are the first contact point between a host or the environment and the bacterium. However, studying the role of exosporial proteins by their inactivation may result in the absence of or defects in other spore proteins. For example, in *B*. *anthracis* mutagenesis of *bxpB* also results in the mislocalisation of BclA [31]. This is a limiting factor when studying the role of proteins using a mutagenesis approach.

In this study, we investigated the *C*. *sordellii* outer spore proteome and identified two proteins, CsA and CsB, that are likely to be associated with these spore components. Insertional inactivation of *csA* and *csB* resulted in the production of spores with structurally defective coat and exosporial layers, with spores of the *csA* mutant also exhibiting altered resistance properties. In comparison to wild-type spores, the *csB* mutation resulted in spores with increased adherence to cervical cell lines, with the cervix being a physiologically relevant anatomical site from which *C*. *sordellii* has been isolated [32, 33]. *C*. *sordellii* enteric disease in animals is well described [9–11, 34]; for this reason, a mouse model of *C*. *sordellii* gastrointestinal infection was developed to determine if CsA and CsB play a role in infection and disease. Using this model, we showed that spores from the *csB* mutant were not cleared as effectively from the gastrointestinal tract and persisted in this niche more effectively than wild-type spores. This study is the first to characterise *C*. *sordellii* outer spore proteins *via* the construction of mutants. The development of a gastrointestinal model of *C*. *sordellii* infection, in which spores are administered to reflect what likely occurs in a naturally occurring infection, has allowed the role of *C*. *sordellii* outer spore proteins to be examined in the context of the host.

## Results

### *C*. *sordellii* outer spore proteins identified by mass spectrometry

To determine the protein composition of the *C*. *sordellii* outer spore, proteins were chemically extracted from the spores of strain ATCC9714 using an alkaline solution without SDS as described in the materials and methods. The absence of SDS prevents the co-extraction of coat proteins with the exosporial proteins [35] and this method has previously been used in *B*. *anthracis* and *B*. *cereus* to identify the exosporial proteins BclA and ExsM [35, 36]. Following the extraction procedure, spores were visualized using transmission electron microscopy (TEM) to confirm the removal of the exosporium without disruption of the rest of the spore, in line with procedures performed by Redmond et al [37]. Most of the exosporium appears to have been removed with the treatment (S1 Fig). Mass spectrometry was then used to determine the identity of the proteins in the extract (S1 Table) and these proteins were assigned to six specific categories (S2 Table) based on categories previously used in the literature [29, 38]. *C*. *sordellii* proteins that were orthologues of proteins identified in the *C*. *difficile* exosporium were assigned to the same categories as those assigned to *C*. *difficile* [29], which included uncharacterized proteins H477_3144 and H477_2973. Although several proteins did not have orthologs in *C*. *difficile*, exosporial proteins of the same name were identified in previous studies [29, 38] and were classified as follows: peptidase M20/M25/M40 family proteins (H477_0435 and H477_0436) were classified according to the peptidase M20 family protein identified in the *C*. *difficile* spore coat [38]; reverse rubrerythrin-1 (H477_0313 and H477_0314) were classified according to rubrerythrin proteins identified in the *C*. *difficile* exosporium spore coat [29, 38]; the manganese-containing catalase family protein (H477_3486) was placed in the ‘spore assembly’ group together with the manganese containing catalase family protein (H477_3485) that showed homology to CotD of *C*. *difficile* [29]. The category assignment of several other proteins was determined with the aid of additional published literature, as follows: putative peptidoglycan binding domain protein (H477_0372) has a SpoIID and peptidoglycan binding domain and may therefore be involved in the degradation of the cortex upon germination [39]; small, acid-soluble spore protein (SASP) beta (H477_4660) was identified in the exosporium of *B*. *anthracis* [40], SASPs protect the DNA of the spore and are typically found in the spore core [2, 40]; coat F domain protein (H477_1527) was identified in the *C*. *difficile* exosporium but its role remains unknown [2]; putative amidase domain protein (H477_1207), cupin domain protein (H477_1872) and fascin domain protein (H477_5266) have not to our knowledge been identified in spores of other species and no putative role could be deduced from the literature. All proteins labeled as ‘putative uncharacterized proteins’ and listed in the ‘unknown putative role’ category either had homology to uncharacterised proteins in the *C*. *difficile* or *B*. *cereus* proteomes or did not show homology to any protein in these species. *C*. *sordellii* proteins categorized as cytosolic proteins were done so because these proteins were the same or similar in name to the cytosolic proteins found in *C*. *difficile* or *B*. *cereus* [29, 38]. Cytosolic proteins identified in the exosporium are likely to be mother cell proteins that were trapped in the exosporium during its formation [29].

### Identification of the CsA and CsB proteins

Two of the uncharacterised *C*. *sordellii* outer spore proteins identified in this study, H477_3144 and H477_4099, showed high peptide sequence coverage in the mass spectrometry analysis and were therefore chosen for further analysis. Specifically, 52 and 21 peptides were identified for protein H477_3144 and 23 and 29 peptides identified for H477_4099, respectively, in each of the biological replicates (S1 Table, S2 Table). Analysis of the upstream genome regions of H477_3144 and H477_4099 identified two other putative uncharacterized proteins (H477_3145 and H477_4098, respectively). Sanger DNA sequencing of these regions confirmed errors in the published sequences of gene H477_3145 (missing nucleotide at position 372) and gene H477_4098 (missing nucleotide at position 432) that resulted in a premature stop codon in both sequences [41]. When these errors are corrected, it appears that H477_3144 and H477_3145, together with the intergenic region, are expressed as a single polypeptide of approximately 45 kDa, designated protein *C*. *sordellii*-A (CsA). Similarly, H477_4098 and H477_4099, together with the intergenic region, were also deemed to encode a single polypeptide of approximately 43 kDa, designated protein *C*. *sordellii*-B (CsB). The gene and protein sequences of CsA and CsB are provided in the supporting information (S1 Appendix).

**Fig 1:**
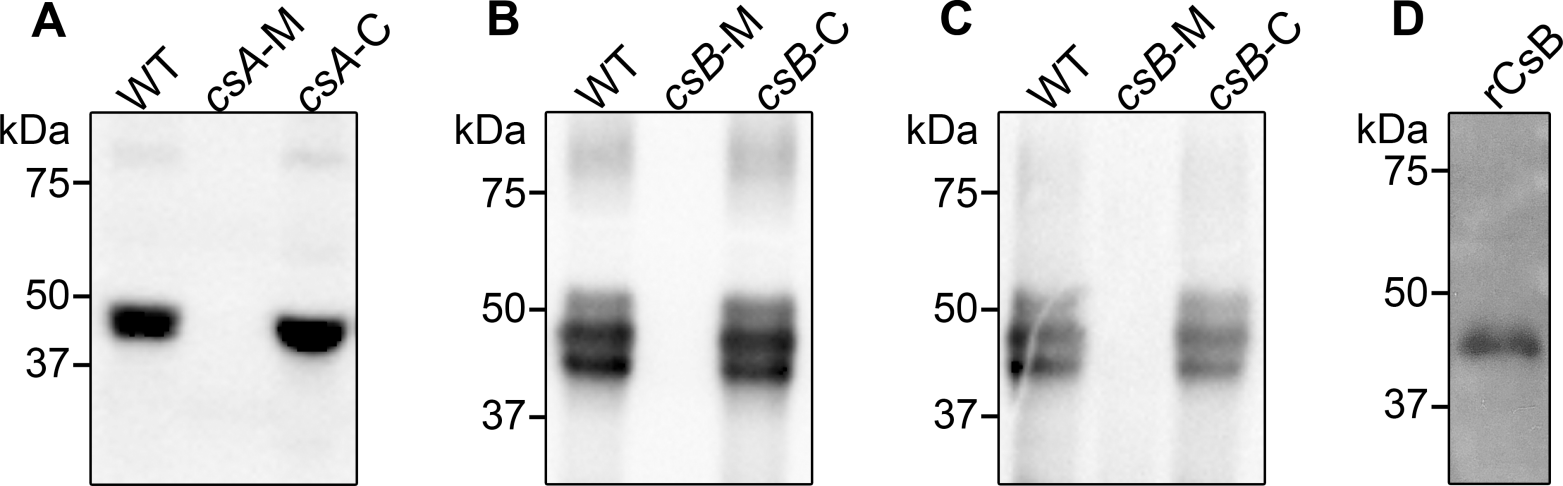
Western blot analysis of CSA and CSB. Exosporial extracts probed with anti-CsA (A), anti-CsB (B) and anti-whole spore antibodies (C) and recombinant CsB probed with anti-whole spore antibodies (D). Expected molecular weights of CsA, CsB and recombinant CsB are 45 kDa, 43 kDa and 44 kDa, respectively. Multiple protein fragments in the wild-type exosporial extracts indicate the presence of multiple protein species for CsA (A) and CsB (B). All protein bands are absent in the *csA* mutant and *csB* mutant with expression restored in the *csA* complemented and *csB* complemented strains. Western blot analysis using whole spore antibodies (C, D) indicates that CsB is an immunogenic protein. Wild-type, WT; *csA* mutant, *csA*-M; *csA* mutant complemented, *csA*-C; *csB* mutant, *csB*-M; *csB* mutant complemented, *csB*-C; recombinant CsB, rCsB.

To confirm that the proteins were produced as single polypeptides, we performed Western blot analysis using recombinantly expressed CsA and CsB with protein-specific antibodies and showed that CsA and CsB are expressed and can be visualised at the predicted sizes (S2A and S2B Fig). The recombinant proteins were expressed in *Escherichia coli* with a C-terminus HIS tag resulting in a 46 kDa and 44 kDa protein for recombinant CsA and recombinant CsB, respectively. Western blot analysis on wild-type exosporial extracts using the CsA-specific antibodies showed the presence of one dominant protein species of about 45 kDa (Fig 1A lane 1). Western blot analysis on wild-type exosporial extracts using the CsB-specific antibodies showed the presence of either two or three dominant protein species (Fig 1B lane 1, S2C Fig). The lower protein band present when three protein species were seen is at approximately 43 kDa, which is the expected size of CsB (Fig 1B lane 1). The middle protein band is approximately 45 kDa and the upper protein band is approximately 50 kDa (Fig 1B lane 1). The presence of multiple protein species for CsB indicates that the protein may be post-translationally modified. Note that there are no alternative start sites in the gene sequence that would result in the production of the other CsB protein species detected.

Given that the *C*. *difficile* protein CdeC [30] showed homology to H477_3144 in a BLAST search against the *C*. *sordellii* genome (S2 Table), we aligned the new full length CsA to CdeC using the BLASTP suite-2sequences program (parameters: maximum identity ≥ 30%, *e*-value of ≤ 0.005%) and showed that the two proteins are homologous (42% sequence identity) (http://blast.ncbi.nlm.nih.gov/Blast.cgi) [42]. The CdeC protein is a 42 kDa cysteine rich protein (cysteine residue content is 8%) [30]. CsA is therefore slightly longer than CdeC, with small insertions observed predominantly in the N- and C-terminus of the protein. CsA contains 8.5% cysteine residues, also making it a cysteine rich protein. Analysis using the Fold and Function Assignment server (FFAS, http://ffas.sanfordburnham.org [43] failed to identify any known protein folds, nor were any conserved domains identified. No transmembrane domains were identified using TMpred (https://embnet.vital-it.ch/software/TMPRED_form.html) [44]. Blast and FFAS analysis of CsB showed that this protein did not share homology with any characterised protein. The BLAST analysis of the CsB protein did identify homologs in *Clostridium bifermentans*, *Paraclostridium benzoelyticum* and *Romboutsia ilealis*, however, these homologs are also annotated as hypothetical proteins [42]. The cysteine content of CsB is not as high as CsA (only 7.9%) but this protein does have a high proline content (10.3%). Comparison of the sequences of CsA to CsB shows that the two proteins share only 17.8% sequence identity.

### Construction of *csA* and *csB* mutant strains and complemented derivatives

The genes encoding CsA and CsB were insertionally inactivated using targetron technology and the mutants confirmed by PCR and Southern blotting (S3 Fig) using *csA-* (S3A Fig), *csB*- (S3B Fig) and intron-specific (S3C Fig) probes. As expected, the *csA*-specific probe detected a band of approximately 2.3 kb and 4.1 kb for the wild-type and the *csA* mutant, respectively. When the *csB*-specific probe was used, a band of approximately 3.2 kb and 5.0 kb was detected for the wild-type and the *csB* mutant, respectively. These size differences correspond to the inserted targetron element (approximately 1.8 kb). The intron-specific probe detected a single band for the mutant strains, confirming the integration of the targetron element. As expected, no intron-specific band was detected in the wild-type DNA.

Complementation of the mutants was achieved *via* homologous recombination through a markerless double cross-over recombination event designed to remove the targetron element from the mutant strains. These revertants were confirmed by Southern blotting (S3 Fig). As expected, Southern hybridization using the *csA*- and *csB*-specific probes detected bands of approximately 2.3 kb and 3.2 kb for the *csA* complemented and *csB* complemented strains, respectively. In addition, no bands were detected when DNA from the complemented strains was probed with the intron-specific fragment, indicating the loss of the targetron elements. Western blot analysis on the exosporial proteins from the wild-type and mutant strains, using CsA- and CsB-specific antibodies, showed that CsA and CsB were absent in the respective mutants (Fig 1A and 1B lane 2) with other exosporial proteins still being produced as detected by sodium dodecyl sulfate gel electrophoresis (SDS-PAGE) (S2D Fig). Complementation successfully restored the proteins in their respective strains (Fig 1A and 1B lane 3). As spore formation is a highly regulated process, complementation was carried out *in cis* for the *csA* and *csB* mutants to retain appropriate regulatory control of the genes.

### *csA* and *csB* mutant spores have structural abnormalities

**Fig 2:**
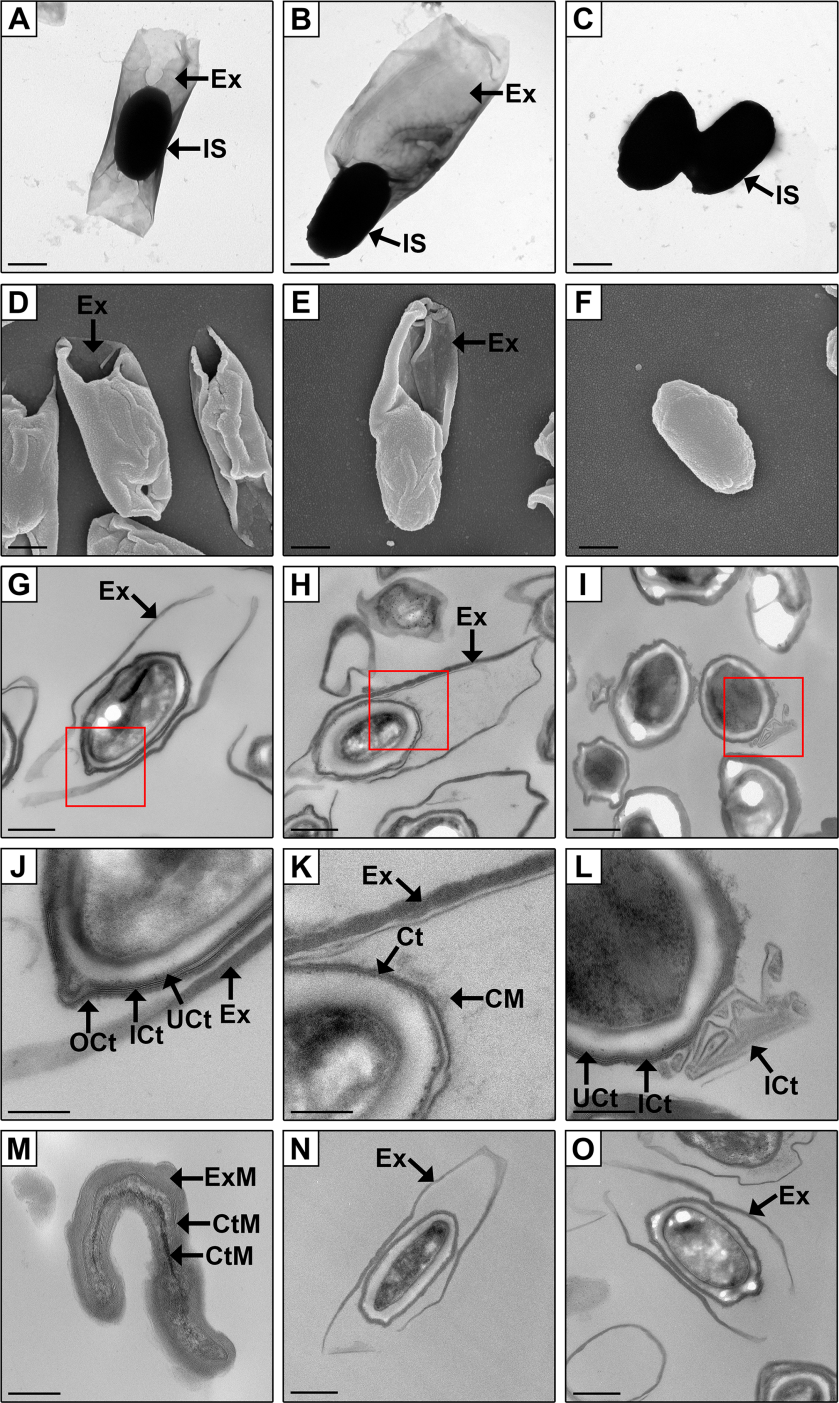
Electron microscopy imaging shows abnormalities in the exosporium and coat structures of the spore mutants. Whole spores were imaged by Transmission Electron Microscopy (TEM) (A-C) and Scanning Electron Microscopy (SEM) (D-F). Spore sections were imaged by TEM (G-O). Images J-L are magnified views of the red boxed areas shown in images G-I, respectively. Wild-type spores (A, D, G, J), *csA* mutant spores (B, E, H, K), *csB* mutant spores (C, F, I, L), detached exosporial and coat material present in the *csB* mutant spore sample (M), complemented *csA* mutant spores (N), complemented *csB* mutant spores (O). In the wild-type strain the inner spore (IS) is centralised within the exosporium (EX). The spore coat (Ct) forms part of the inner spore (IS) and in the wild-type strain is composed of three layers (OCt, ICt, UCt). Ex, exosporium; IS, inner spore; Ct, coat; OCt, outer coat; ICt, inner coat; UCt, under coat; ExM, exosporial material; CtM, undefined coat material. Scale bar: images A-I, N-O = 0.5 μm; images J-M = 0.2 μm.

Visualisation by electron microscopy showed that the exosporium and inner spore of the *csA* and *csB* mutants differed to the wild-type (Fig 2). Spores of the wild-type strain had an inner spore composed of multiple distinct layers, and the inner spore was located within the centre of a baggy exosporium (Fig 2A, 2D, 2G and 2J, S4A Fig). By comparison, *csA* mutant spores had an inner spore that was positioned towards one pole of the exosporium (Fig 2B, 2E and 2H, S4B Fig), with 37 out of 40 whole spores analysed by TEM exhibiting this phenotype in comparison to 3 out of 40 wild-type whole spores analysed (S4D Fig). Furthermore, the inner spore layers of the *csA* mutant did not appear to be as distinct as those of the wild-type strain (observed in 10 out of 10 spores) (Fig 2J and 2K), which may indicate an abnormality in these structures. Electron dense bodies, which appeared to originate from the spore coat, were commonly observed in the interspace of the *csA* mutant (8 out of 10 spores observed) (indicated by CM, Fig 2K), but not in any of the wild-type spores (0 out of 10 spores observed), and may indicate spore coat instability in the mutant. The length and width of *csA* mutant spores (including the inner spore and the exosporium) appeared to be the same as those produced by the wild-type strain (S4F–S4H Fig).

Unlike the *csA* mutant spores, *csB* spores did not have an attached exosporium (Fig 2C, 2F and 2I), and this was observed in all spores analysed by TEM (n = 40). However, putative detached exosporia were seen in the *csB* spore samples (Fig 2M, S4E Fig), and, as discussed earlier, exosporial proteins could be detected by SDS-PAGE (S2D Fig), suggesting that although *csB* mutant spores produce an exosporium it is not correctly tethered to the remainder of the spore. Spores of the *csB* mutant had distinct inner spore layers, but the coat was structurally aberrant in all spores observed (minimum of 10 out of 10 observed). Specifically, the outer spore coat appeared to be absent from spores of the *csB* mutant, and the inner spore coat appeared to be partially mislocalised in some spores (Fig 2L). In addition, fragments presumably composed of coat material were observed in spores of the *csB* mutant and may have sloughed off the inner spore (indicated by CtM, Fig 2M). The lengths and widths of the inner spores remained unchanged between the wild-type and mutant spores (S4F and S4G Fig). Note that the mutants exhibited a wild-type spore morphology when complemented *in cis* with the intact gene (Fig 2N and 2O).

### CsB is an immunogenic protein

Since CsA and CsB appear to be located on the spore surface, we investigated if these proteins were immunogenic by raising antibodies against whole wild-type spores in rabbits. Western blot analysis on exosporial protein extracts from the wild-type, *csA* and *csB* mutant strains was performed using these antibodies (Fig 1C, S2E Fig). Though spores of the *csB* mutant do not have an attached exosporium, detached exosporial material is visible in the spore sample (Fig 2M, S4E Fig) and proteins from this material were extracted for the analysis. Prominent protein bands were detected in the wild-type and *csA* mutant strains but were absent in the *csB* mutant strain (Fig 1C, S2E Fig). These protein bands were of a similar molecular weight to those previously detected by Western blotting using CsB-specific antibodies against the wild-type exosporial extract (Fig 1B lane 1, S2C Fig). We performed the same analysis using recombinant CsB and a protein of approximately 44 kDa in size was detected, as expected (Fig 1D). These results suggest that CsB is an immunogenic protein in rabbits.

### Sporulation efficiency of the *csA* mutant differs to wild-type

**Fig 3:**
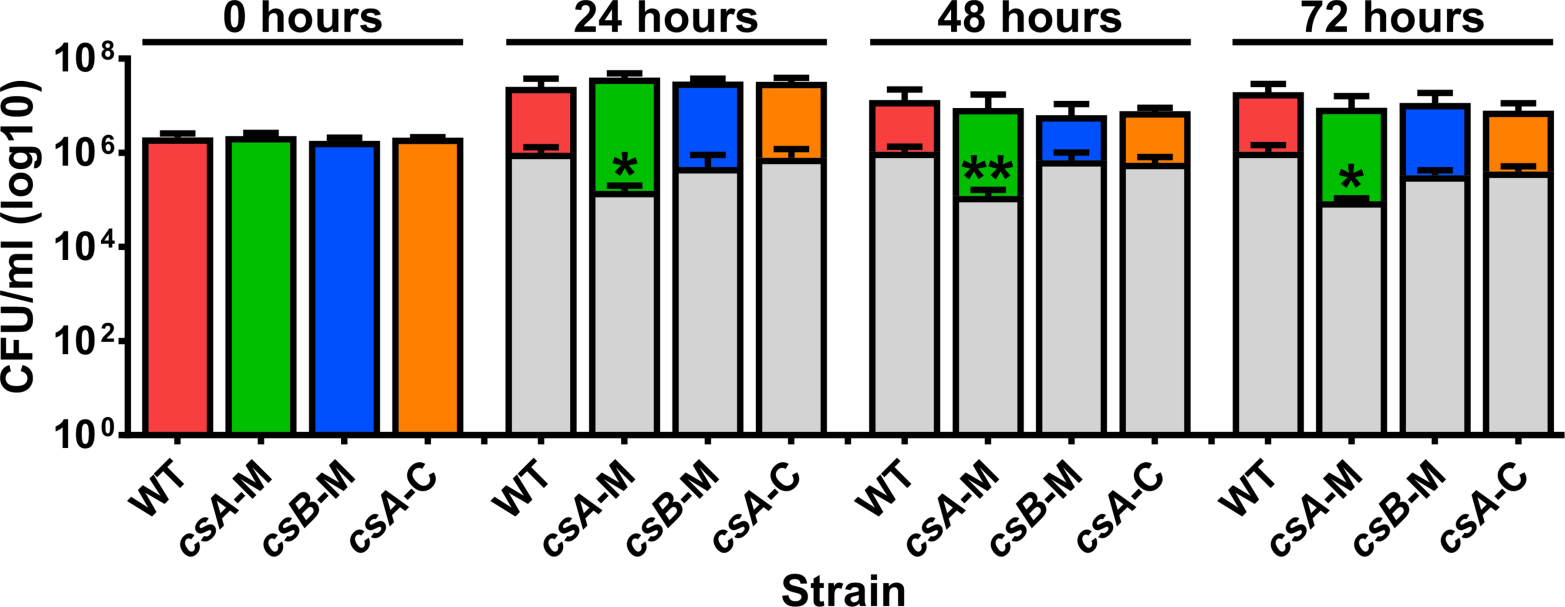
The *csA* mutant produces less viable spores than the wild-type strain. Strains were inoculated into liquid sporulation media with the total cell count (spores and vegetative bacteria) (coloured bars) and spore count alone (grey bars) determined every 24 hours. At each time point all strains show similar total cell counts, however the *csA* mutant produces less spores than the wild-type strain. The results represent the average of four independent experiments and error bars represent standard error of the mean. Asterisks indicate statistical difference in comparison to WT at *, *p* ≤ 0.05; **, *p* ≤ 0.01. Wild-type, WT; *csA* mutant, *csA*-M; *csB* mutant, *csB*-M; *csA* mutant complemented, *csA*-C.

All strains were tested for their ability to produce viable spores in liquid sporulation media over a 72 hour period. There was no difference in the total cell counts (spores and vegetative bacteria) between the strains at any time (Fig 3). Additionally, for each strain, there was no significant increase in the number of spores produced at 24 hours compared to 72 hours post-inoculation, indicating that sporulation was complete for all strains within 24 hours. However, the *csA* mutant produced 6- to 11-fold less viable spores (84%-91% reduction) compared to the wild-type strain at each time point post-inoculation (*p* = 0.0087–0.0317).

### The *csA* and *csB* mutations do not affect the colony forming efficiency of untreated spores but affect the colony forming efficiency of *csA* mutant spores when treated with heat or chemicals

**Fig 4:**
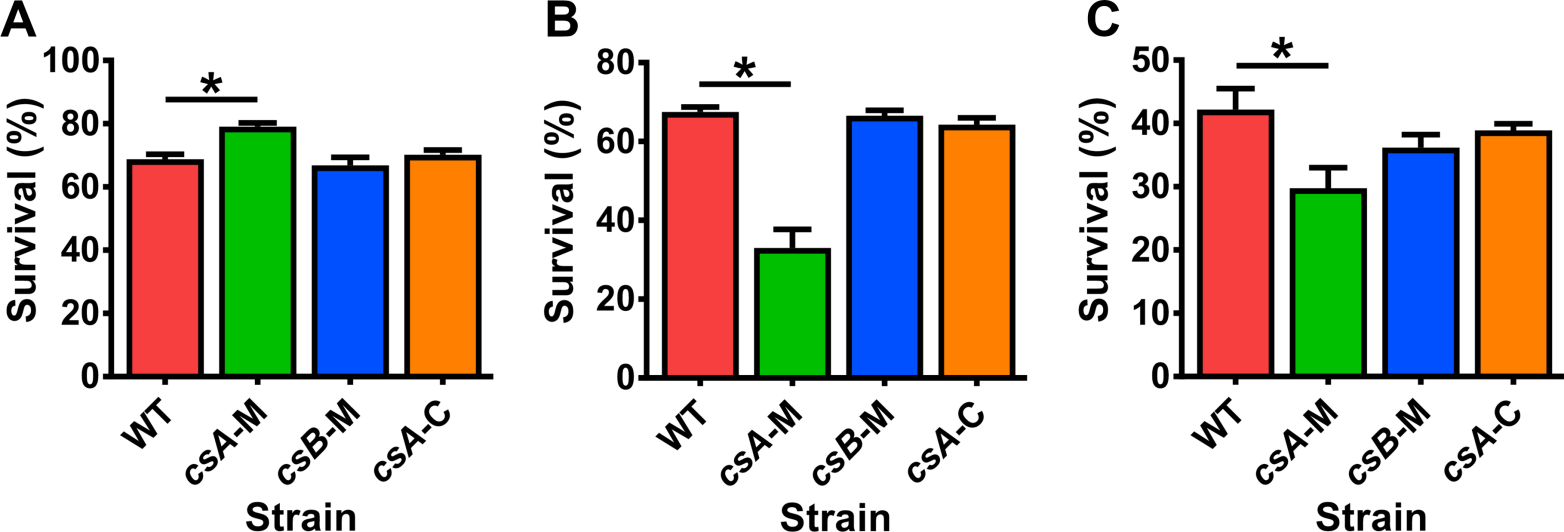
Spores of the *csA* mutant have altered heat and chemicals resistance properties compared to wild-type spores. Spores were treated to 75°C heat (A), 0.5 M hydrochloric acid (B) and 0.15 M sodium hydroxide (C) for 30 minutes after which the spores were incubated on HIS agar to determine the percentage of spore survival. The results represent the average of four independent experiments and error bars represent standard error of the mean. Asterisks indicate statistical difference at *p* ≤ 0.05. Wild-type, WT; *csA* mutant, *csA*-M; *csB*mutant, *csB-*M; *csA* mutant complemented, *csA*-C.

The colony forming efficiency of the spores was compared by plating a standardised number of purified spores, as determined by counting spores with a haemocytometer, onto solid media followed by growth at 37°C. No differences in colony forming efficiencies were observed between the strains (S5A Fig). The spores from all strains were then examined for their ability to withstand high temperatures and various chemical treatments. Spores of the *csA* mutant showed an 15% increase in colony forming efficiency when incubated at 75°C (Fig 4A), a 51% decrease in colony forming efficiency when incubated with hydrochloric acid (Fig 4B) and a 29% decrease in colony forming efficiency when incubated with sodium hydroxide (Fig 4C) when compared to wild-type spores (*p* = 0.0286). Spores of the *csB* mutant did not show sensitivity to any of these treatments in comparison to wild-type spores. Spores of all strains were also incubated with 1 mg/ml lysozyme and 80% ethanol, however, no differences in spore colony forming efficiency were detected between the strains following these treatments (S5B and S5C Fig). Spore colony forming efficiency of the *csA* mutant in response to heat, hydrochloric acid and sodium hydroxide returned to wild-type levels upon complemention (Fig 4).

### *csB* mutant spores display enhanced adherence to cervical cells compared to wild-type spores

**Fig 5:**
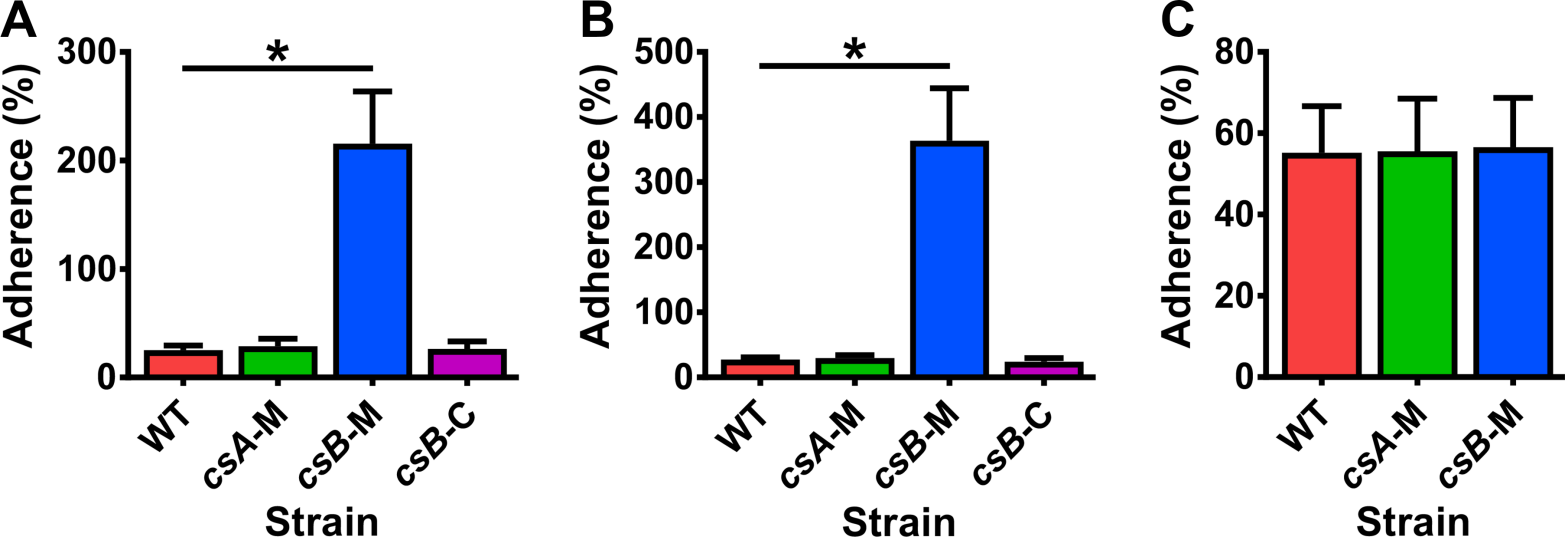
Spores of the *csB* mutant show increased adherence to cervical but not vaginal cells compared to wild-type spores. Cervical cell lines Ect1/E6E7 (A) and End1/E6E7 (B) and vaginal cell line Vk2/E6E7 (C) were incubated with spores for 3 hours, washed to remove unbound spores and the number of bound spores determined. The results represent the average of four independent experiments and error bars represent standard error of the mean. Asterisks indicate statistical difference at *p* ≤ 0.05. Wild-type, WT; *csA* mutant, *csA*-M; *csB* mutant, *csB*-M; *csB* mutant complemented, *csB*-C.

*C*. *sordellii* causes necrosis and oedema of the female reproductive tract post-birth or post-abortion, perhaps as a result of vaginal tearing which may allow contaminating bacteria to ascend a dilated cervix [12]. *C*. *sordellii* has also been isolated from the cervix of women presenting with pelvic infections [32, 33] and one study identified 0.2% of healthy women as asymptomatic vaginal carriers [8]. Here, human cervical (Ect1/E6E7 and End1/E6E7) and vaginal (VK2/E6E7) cell lines were used to investigate if wild-type spores adhered to these physiologically relevant cell lines and if spore abnormalities in the *csA* and *csB* mutants altered their adherence properties (Fig 5). The results showed that 55% of spores isolated from the wild-type and *csA* mutant adhered to the Ect1/E6E7 and End1/E6E7 cell lines 3 hours post-infection (Fig 5A and 5B). However, spores of the *csB* mutant were more adherent with a 9- fold and 13- fold increase in adherence to the Ect1/E6E7 and End1/E6E7 cell lines compared to wild-type spores, respectively (*p* = 0.0286) with adherence returning to wild-type levels upon complementation. No difference was detected between the different strains on the VK2/E6E7 cell line, with 55% of spores found to adhere three hours post infection (Fig 5C).

### *csB* mutant spores persist in the gastrointestinal tract of infected mice

*C*. *sordellii* has been isolated from the gastrointestinal tract of humans and animals and causes enteric disease in animals [8–11]. In a similar way to *C*. *difficile*, it has been hypothesised that changes in the gastrointestinal tract environment or microbiome may induce susceptibility to *C*. *sordellii* infection and that the infection source can be internal or external [9–11, 34]. Although *C*. *sordellii* soft tissue infection models have been developed [45–47], a gastrointestinal tract infection model has not been reported. Furthermore, despite the importance of spores in the infectious cycle, previous *C*. *sordellii* animal models have administered vegetative cells and not spores to establish infection in animals and spores have not been monitored during these infections [45–47].

**Fig 6:**
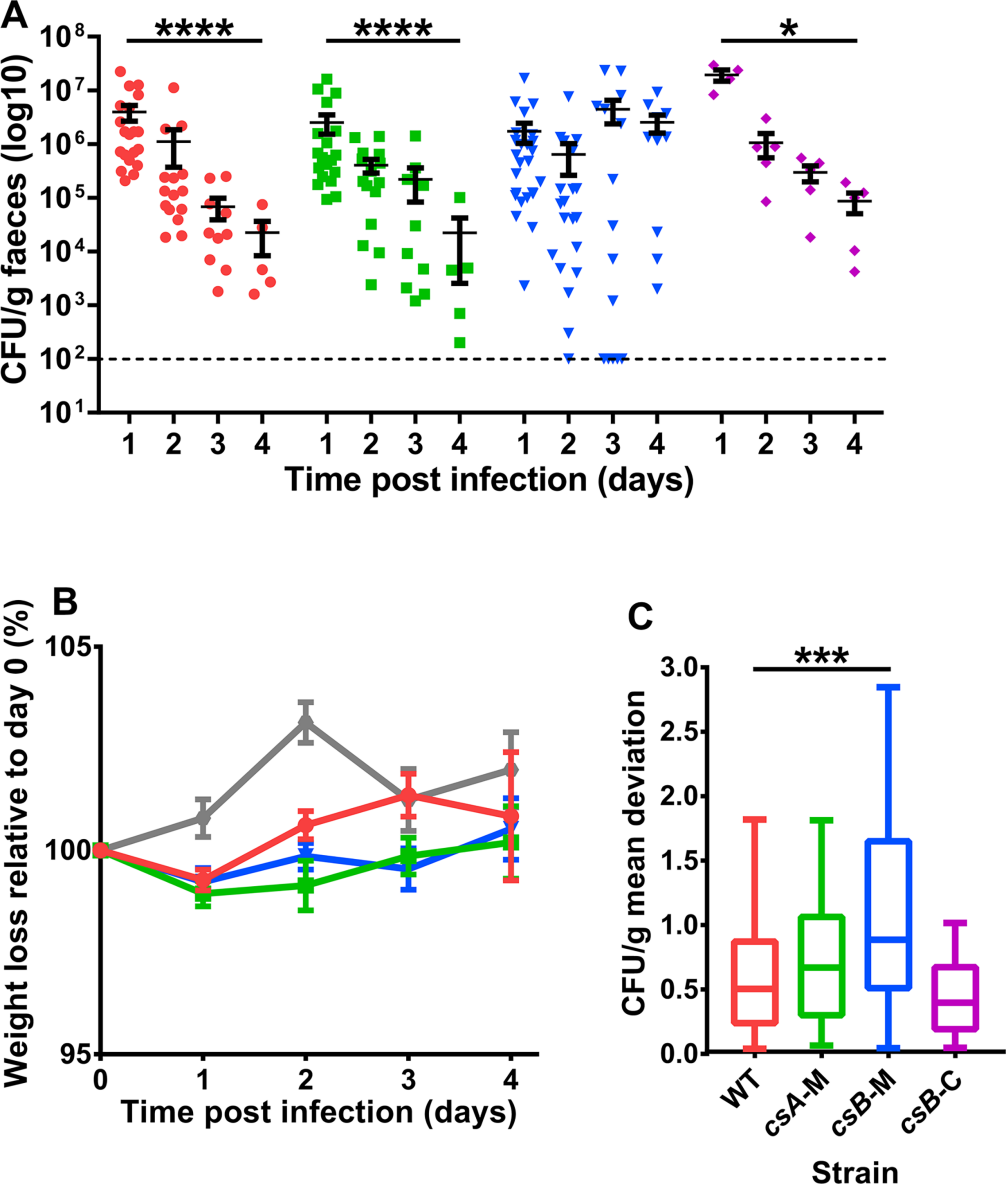
The *csB* mutant shows increased colonisation in a mouse model of gastrointestinal tract infection, but no difference in weight loss, compared to the wild-type strain. Mice orally infected with spores from the *csB* mutant show high levels of spores shed from the gastrointestinal tract for the duration of the experiment (A). Mice were administered either wild-type (red circle), *csA* mutant (green square), *csB* mutant (blue arrowhead) or *csB* complemented (purple diamond) spores. Faecal spore load was determined for each mouse every day until 4 days post infection and is presented here with each point representing a single mouse. The results represent the average of at least four mice and error bars represent standard error of the mean. The limit of detection is shown as a dashed line (A). Mice administered wild-type spores (red) showed no difference in weight loss compared to mice administered *csA* (green) or *csB* (blue) spores or to uninfected mice (grey). The results represent the average of at least five mice and error bars represent standard error of the mean (B). The variability in faecal spore load between mice administered with the same strain was calculated by determining the deviation in faecal spore load of each mouse in comparison to the mean faecal spore load of mice administered with the same strain and is presented here as box plots with Tukey whiskers (C). Asterisks indicate statistical difference at *, *p* ≤ 0.05; **, *p* ≤ 0.01; ****, *p* ≤ 0.0001. Wild-type, WT; *csA* mutant, *csA*-M; *csB* mutant, *csB*-M; *csB* mutant complemented, *csB*-C.

To investigate the role of wild-type and mutant *C*. *sordellii* spores in the gastrointestinal tract, a mouse infection model was established. In this model, mice were administered antibiotics to disrupt their gastrointestinal tract microbiota before being orally gavaged with *C*. *sordellii* spores (a minimum of five mice were orally gavaged with each strain). Mice were then monitored for spore shedding in their faeces every day post infection, as a surrogate measure of *C*. *sordellii* colonisation. With the wild-type strain, the mice had high numbers of faecal spores initially (mean of 4 x 10^6^ at 1 day post-infection) with the number of spores decreasing over the duration of the experiment (mean of 2 x 10^4^ at 4 days post-infection) (Fig 6A). These results suggest that strong colonisation occurs post-infection after which wild-type levels of *C*. *sordellii* are significantly reduced in the gastrointestinal tract. In addition, mice administered wild-type spores did not display disease symptoms of weight loss (Fig 6B), diarrhea and behavioral changes in comparison to uninfected mice.

Colonisation of the *csA* and *csB* mutants was examined in the mouse infection model in comparison to the wild-type strain. Mice were infected with spores from the wild-type, mutant or complemented strains and faecal spore numbers monitored every day post-infection for the duration of the experiment. Mice infected with *csA* mutant spores had similar colonisation patterns to mice infected with wild-type spores. Spore shedding from animals infected with these strains steadily decreased over time, resulting in a significant decrease in faecal spore numbers at 4 days (mean of 2 x 10^4^ for wild-type and *csA*) compared to 1 day (mean of 4 x 10^6^ and 3 x 10^6^ for wild-type and *csA*, respectively) post-infection (*p* = < 0.0001) (Fig 6A). By contrast, mice infected with *csB* mutant spores did not have a significant decrease in faecal spore numbers at 4 days (mean of 3 x 10^6^) compared to 1 day (mean of 2 x 10^6^) post-infection. Colonisation levels were restored to those of the wild-type strain when *csB* was complemented *in cis* (Fig 6A). The *csA* complemented strain was not included in this analysis as there was no difference in the colonisation pattern in mice infected with wild-type and *csA* mutant spores. Furthermore, the mutations do not appear to affect the pathogenesis of *C*. *sordellii* in the mouse infection model as no significant differences in the disease symptoms of weight loss (Fig 6B), diarrhea or behavioural changes were observed between mice infected with mutant or wild-type strains.

To determine the variability in spore shedding between mice infected with each strain, the shedding values at every time point for a particular strain were combined and the mean was calculated. The deviation of each shedding value from this mean was determined and a Tukey post-hoc test was performed. This analysis demonstrated that mice infected with the *csB* mutant exhibited a greater variability between mice in the level of faecal spore shedding compared to mice infected with the wild-type, *csA* mutant and *csB* complemented strains (Fig 6C). Finally, note that growth assays showed that growth between the *C*. *sordellii* strains was similar (S6 Fig) and therefore, these parameters were not responsible for the variable colonisation levels of the strains in the mouse model.

## Discussion

*C*. *sordellii* is a human and animal pathogen and the spores produced by this bacterium are important for infection [12, 15, 16, 34]. We recently characterised the *C*. *sordellii* spore structure and showed that the outer spore layers consist of a coat which is surrounded by a baggy exosporium [18]. Spore surface proteins are likely to be important for early host interactions and disease and may be important for spore fitness within the host. Mutagenesis of exosporial proteins, such as CdeC in *C*. *difficile* and superoxide dismutases and alanine racemase in *B*. *anthracis*, resulted in spore structural abnormalities, disease attenuation in a mouse infection model and premature germination, respectively [30, 48, 49]. However, *C*. *sordellii* spores are structurally dissimilar to spores of *C*. *difficile* and *B*. *anthracis*. Therefore, to examine the composition and function of the *C*. *sordellii* exosporium, we performed mass spectrometry analysis on this extracted fraction and identified numerous proteins (S1 Table, S2 Table). Two of these proteins, CsA and CsB, were characterised further in this study. CsA shared homology to the *C*. *difficile* protein CdeC whereas CsB did not have homology to characterised proteins from any other species. Here, we showed that CsA and CsB are important for the correct assembly of the *C*. *sordelli* spore and that *csB* mutant spores appear to be more adherent to human cervical cell lines and to persist in a mouse gastrointestinal infection model.

Mutagenesis of the *csA* and *csB* genes resulted in structurally aberrant spores and mutagenesis of *csB* also resulted in spores with altered resistance properties. While wild-type *C*. *sordellii* spores had an exosporium that wraps around a centrally located inner spore (Fig 2A, 2D and 2G), *csA* mutant spores had an inner spore located towards one pole of the exosporium (Fig 2B, 2E and 2H). Thus, it is possible that CsA anchors or stabilises the position of the inner spore within the exosporium. No filaments have been observed connecting the exosporium to the inner spore [18] but it is likely that protein-protein interactions hold the structures together and CsA may play a role in these interactions. Although the spores of the *csA* mutant have an inner spore that is spatially disorientated, the exosporium appears to be present and associated with the inner spore. This is unlike spores isolated from a *C*. *difficile cdeC* mutant, where the exosporium was either partially or completely absent from the inner spore [30]. This result suggests that the CsA and CdeC proteins appear to serve a different structural or functional role within each species despite their homology to one another.

Spores isolated from the *csB* mutant appear to lack an exosporium (Fig 2C, 2F and 2I), however, detached exosporial material was visible in the spore samples (Figs 2M and S4E). Importantly, *csB* mutant spores did not show any abnormalities in spore colony forming efficiencies (S5A Fig), which requires spores to complete all stages of germination [50], even though the exosporium plays a role in regulating germination in other species. An examples of the exosporium playing a role in germination is seen in *B*. *anthracis* where the exosporial enzyme alanine racemase converts the germinant L-alanine to D-alanine, which inhibits germination [48]. Mutagenesis of the gene encoding alanine racemase resulted in premature germination of the developing spore within the mother cell and more efficient germination of the mature spore in comparison to wild-type spores [48]. In spores of *C*. *difficile* the exosporium also appears to play a role in germination. A previous study showed that *C*. *difficile* spores which had varying amounts of the exosporium proteolytically removed had higher germination levels when less of the exosporium was present [51]. In this regard, *C*. *sordellii* appears to be different to *B*. *anthracis* and *C*. *difficile* as the spore colony forming efficiency, and consequently spore germination, were unaffected in the *csB* mutant spores. Alternatively, the detached exosporium present in the spore preparations may be providing the enzymatic functions needed to regulate germination.

As well as an abnormal exosporium, *csA* and *csB* mutant spores appeared to have spore coat defects. Electron dense material within the interspace region of *csA* mutant spores was seen, which may represent fragments of the coat that were dissociated from the rest of the spore (Fig 2K). An outer spore coat was absent in all *csB* mutant spores and the inner coat appeared partially mislocalised in some of these spores (Fig 2L). These results suggest that the *csA* and *csB* mutations destabilise the spore coat layers. Mutagenesis of exosporial proteins in other species, such as *cdeC* in *C*. *difficile*, have also resulted in structural abnormalities to the exosporium and coat [30]. The coat is believed to protect spores from lysozyme [52], however, *csA* and *csB* mutant spores were as resistant to lysozyme treatment as wild-type spores (S5B Fig), suggesting that the coat present in these mutants remains impermeable to lysozyme or that another mechanism is conferring this resistance phenotype. In comparison to *csA* mutant spores, *C*. *difficile cdeC* mutant spores appeared to have all coat layers intact but the layers differed in thickness and spores differed in sensitivity to lysozyme in comparison to wild-type spores [30]. In a similar way to *csB* mutant spores, *C*. *difficile cotA* and *B*. *subtilis cotE* mutants also lacked an outer spore coat, however, in contrast to *csB* spores, these spores had increased sensitivity to lysozyme compared to the wild-type and the *cotA* mutants also had increased sensitivity to ethanol [53, 54].

Additional inner spore abnormalities were also detected in *csA* mutant spores. Spores of the *csA* mutant were more sensitive to hydrochloric acid compared to wild-type spores (Fig 4B). Spores of the *csA* mutant also had increased sensitivity to sodium hydroxide (Fig 4C). This result may reflect defects in cortex hydrolysis since treating *B*. *subtilis* spores with sodium hydroxide appeared to inactivate cortex lytic enzymes, thus preventing the complete germination of spores [55]. In *B*. *subtilis*, spores treated with hydrochloric acid subsequently ruptured, suggesting that hydrochloric acid affects the permeability barrier of these spores [55]. The inner membrane of *B*. *subtilis* spores may be important in maintaining this permeability barrier and may help to keep the spores dehydrated and resistant to hydrochloric acid, heat and ethanol [55]. Although *csA* mutant spores had greater sensitivity to hydrochloric acid, they were more resistant to heat and did not show an increased sensitivity to ethanol in comparison to wild-type spores (Fig 4A and 4B, S5C Fig). Similarly, *csA* mutant spores also differed to *C*. *difficile cdeC* mutant spores since *cdeC* mutant spores were more sensitive to heat and ethanol compared to wild-type spores [30]. These results suggest that the spore structures of *C*. *sordellii*, *B*. *subtilis* and *C*. *difficile* may serve different functions and may therefore respond in different ways to the same treatments.

In the *csB* mutant, the inner coat forms the outermost layer of the spore, not the outer coat as found in wild-type spores. It is therefore likely that spore surface properties are altered in this mutant. In support of this hypothesis, *csB* mutant spores displayed a greater level of adherence to ectocervical and endocervical cells, but not vaginal cells, compared to wild-type spores (Fig 5), suggesting that the inner coat surface has a high affinity for cervical cells. By comparison, *csA* mutant spores displayed the same adherence levels as wild-type spores (Fig 5). It is possible that the exosporium masks adhesins in the coat that are specific to cervical cells and, consequently, these adhesins are exposed in the *csB* mutant spores. Alternatively, the exosporium may be responsible for specific binding of spores to cervical cells and, as a result, in the absence of an exosporium the *csB* mutant spores may bind non-specifically to cervical cells. A similar phenomenon has been seen in *cdeC* mutant spores, which are missing the outer layer of the exosporium and show higher levels of adherence to gut epithelial cells compared to wild-type spores [30, 56]. Of note, wild-type spores adhered to both vaginal and cervical cell lines, reflecting the published literature, which reports that *C*. *sordellii* has been isolated from both regions of the female reproductive tract [8, 32, 33].

Spores of the *csB* mutant also persisted in the gastrointestinal tract of infected mice (Fig 6A). In a similar way, mutations in the *C*. *difficile* genes encoding the spore surface proteins BclA2 and BclA3 resulted in strains that displayed increased colonisation in a mouse model of gastrointestinal infection [57]. Conversely, in *B*. *anthracis*, a mutation in the gene encoding a similar spore surface protein, BclA, resulted in a strain with decreased persistence in the host when tested in a pulmonary mouse infection model of anthrax [35, 58]. In addition to the persistence of the *csB* mutant in the gastrointestinal tract, spore shedding from this mutant was more variable between mice challenged with this strain compared to mice challenged with the wild-type strain (Fig 6C). The increased persistence of the *csB* strain may be due to the misassembled spore coat having a greater affinity for the mouse gut epithelium. Given that the spore is less persistent in its native conditions, perhaps the exosporium plays a role in spore transmission. Furthermore, the variation observed in spore shedding for mice challenged with the *csB* mutant may be due to the variable inner coat presentations of *csB* mutant spores, which may have differing affinities for the gut epithelium. Given that there were no differences in sporulation or colony forming abilities between the wild-type and *csB* mutant (Fig 3, S5A Fig), it is unlikely that these factors played a role in the colonisation patterns observed in the mouse gut model. Note that although CsB was found to induce an adaptive immune response in rabbits (Fig 1C and 1D), the innate immune response to this protein was not examined. For this reason, it was not possible to determine if an altered innate response to infection with the *csB* mutant compared to the wild-type plays a role in the persistence of this strain in the infection model. It is possible that the absence of CsB affects the ability of the innate immune system to detect and clear this strain from the gastrointestinal tract in comparison to the wild-type strain, leading to the colonisation and shedding patterns detected. It is unlikely that the adaptive response to this protein plays a role in clearance of the wild-type strain in the *C*. *sordellii* infection model used here because of the short duration of infection (4 days), however, such a response may be important and protective during natural infections.

The outer structures of spores, including the exosporium, are functionally important because they are the point of contact between the spore and the environment. These structures may also be involved in spore resistance, adherence and in the regulation of germination [24, 25, 28, 48]. Despite their importance, the current understanding of outer spore structures and the role of the proteins that compose these structures is limited, especially in the context of *C*. *sordellii*. Here we have shown that the *C*. *sordellii* outer spore proteins CsA and CsB are necessary for the correct assembly of both the inner spore and exosporium. CsA is required for wild-type levels of spore resistance to heat, sodium hydroxide and hydrochloric acid, while CsB is required for normal levels of spore adherence to cervical cells. The absence of CsB also allows the *csB* mutant to persist within an infected host in a gastrointestinal infection model, possibly because of the increased adherence properties of *csB* spores, however, this needs to be investigated further. The difference in structural abnormalities and resistance properties between spores of the *C*. *sordellii csA* mutant and the *C*. *difficile cdeC* mutant illustrates that, despite their seeming homology, spore proteins can have variable functions in different species. This finding is not unexpected based on the structural variability between spores from these two species [18] and has been shown before for the ExsY protein from *B*. *cereus* and *B*. *anthracis* [59, 60]. Importantly, this result highlights the necessity of studying each spore protein in the cognate species from which it originates. Gaining an understanding of outer spore proteins across a broad cross-section of sporulating bacteria is increasing our knowledge of the various roles that they play, particularly in a structural context and in interactions with the infected host.

## Materials and methods

### Bacterial strains, culture conditions and spore production

All strains of *C*. *sordellii* were derivatives of ATCC9714 and cultured as previously described [18] unless otherwise stated. Media were supplemented with one or more of the following antibiotics where appropriate: D-cycloserine (250 μg/ml), erythromycin (10 μg/ml), thiamphenicol (10 μg/ml) or anhydrous tetracycline (50 ng/ml). *C*. *sordellii* spores were prepared and stored as outlined previously [18] and were determined by light microscopy to be >99% free of vegetative cells and debris. *E*. *coli* strains HB101, DH5α and TOP10 were grown in 2x YT media with the growth conditions and strain characteristics described previously [61]. *E*. *coli* strain BL21(C43) [62] was grown in LB media. *E*. *coli* cultures were supplemented with chloramphenicol (30 μg/ml), tetracycline (10 μg/ml) or kanamycin (20 μg/ml) where necessary.

### Identifying *C*. *sordellii* outer spore proteins by mass spectrometry (MS)

Outer spore proteins were prepared from two independent biological spore stocks and MS performed independently on each extract. *C*. *sordellii* outer spore proteins were extracted from purified spores as previously described [35]. Briefly, spores were incubated at 90°C for 15 min in an extraction buffer containing 8 M urea and 2% 2-mercaptoethanol. The sample was then centrifuged at 13,000x *g* for 10 min and the supernatant, containing the extracted proteins, was dialysed against phosphate buffered saline (PBS) (137 mM NaCl, 2.7 mM KCl, 1.4 mM KH_2_PO_4_, 4.3 mM Na_2_HPO_4_) using a molecular weight cut-off between 6000–8000. The proteins were reduced with 25 mM dithiothreitol, alkylated with 55 mM iodoacetic acid and then trypsin digested in a 1:25 trypsin to protein ratio. The peptides were desalted and concentrated using a peptide trap (Michrome peptide Captrap) with 0.1% formic acid and 2% acetonitrile at 8–10 μl min^-1^. The peptides were then eluted from the column in a linear gradient (0%-90% acetonitrile, in 0.1% formic acid) over 80 min using a flow rate of 0.5 μl/min. Each sample was then subjected to positive ion nanoflow electrospray MS (QSTAR, SCIEX at the Australian Proteome Analysis facility), operated in information acquisition mode, and a time-of-flight-MS survey scan was acquired (m/z 380–1600). From the survey scan, the three most intense multiply charged ions (counts ≥25) were selected for MS/MS analysis (m/z 100–1600). Using ProteinPilot version five (SCIEX), MS data was used to search against the *C*. *sordellii* strain ATCC9714 sequence obtained from UniProt (taxonomy ID 1292036) [63]. The protein false discovery rate was set at 1%, and proteins were reported only if at least one unique peptide was identified in both independent biological replicates analysed. Exosporial proteins previously identified in *C*. *difficile* by MS [29] were then used to search for orthologs to the outer spore proteins identified by MS in this study. BLASTP (http://blast.ncbi.nlm.nih.gov/Blast.cgi) was used for this analysis [42] with a maximum identity of ≥ 30% and an *e*-value of ≤ 0.005% to determine orthologs present. The *C*. *sordellii* proteins labeled as ‘putative uncharacterised proteins’ were also searched for orthologs in the proteomes of *C*. *difficile* (taxonomy ID 1496) and *B*. *cereus* (taxonomy ID 1396) to determine any homology to a characterized protein. *C*. *sordellii* exosporial proteins categorised as cytosolic proteins were excluded from this analysis, to be consistent with the proteins classified as exosporial proteins in *C*. *difficile* [29]. The mass spectrometry proteomics data have been deposited to the ProteomeXchange Consortium *via* the PRIDE [64] partner repository with the dataset identifier PXD009235.

### Construction of *C*. *sordellii* ATCC9714 *csA* and *csB* mutants and complemented strains

The *csA* and *csB* mutants were constructed in strain ATCC9714 using targetron technology as previously described [61], with the exception that the plasmid pDLL46 was retargeted to give rise to pDLL57 and pDLL58 for the disruption of *csA* and *csB*, respectively. Plasmid pDLL46 is a derivative of pMTL9361 [65] with the *Hind*III and *Bsr*GI sites removed from *rep* and *lacZa* and an *oriT* from RP4 and Tn916 included in this vector. Plasmids pDLL57 and pDLL58 were then conjugated into *C*. *sordellii* strain ATCC9714. Expression of the targetron element was induced by the addition of anhydrous tetracycline (50 ng/ml) with the targetron element inserting after nucleotide 920 on the antisense strand for *csA* and after nucleotide 742 on the sense strand for *csB*. PCR and Southern blotting were then used to confirm the correct insertion of the targetron element and Western blot analysis using protein specific antibodies was used to confirm the loss of expression of CsA and CsB in each mutant. The *csA* mutant was designated DLL5069 and the *csB* mutant was designated DLL5071.

Complementation of the *csA* and *csB* mutants was performed using a markerless double cross-over homologous recombination system, as previously described [66], to remove the targetron element. Briefly, PCR was used to amplify DNA fragments containing the wild-type *csA* gene (primer DLP551 AGCATGCTGTAGATTTATCTGGCGTTTTACAC and primer DLP552 AAAGACGTCTATGGATCTTCAATACTATTCGACC) or the wild-type *csB* gene (primer DLP556 AAAGACGTCTTTTAATTGGTTCACTCCATGTGTC and primer DLP557 AGCATGCGAAACCTCTACTTCACTAGCATTGT) as well as genomic regions both upstream and downstream of each gene to generate fragment lengths of 2.7 and 2.8 kb, respectively. The fragments were cloned into the *AatII*/*SphI* sites of the clostridial vector pJIR3566 [62]. The correct DNA sequences of the fragments were confirmed by nucleotide sequencing and the plasmids then designated pDLL165 and pDLL167 for *csA* and *csB*, respectively. Conjugative transfer of the appropriate plasmid into DLL5069 or DLL5071 from the donor strain *E*. *coli* HB101 (pVS520) was performed as previously described [61]. Isolates growing on HIS agar supplemented with D-cycloserine and thiamphenicol were passaged in HIS broths for up to five days and then plated onto HIS agar. Individual colonies were tested for their sensitivity to erythromycin which indicated the loss of the targetron element and plasmids pDLL165 or pDLL167. Complementation of the mutations was again confirmed by PCR, Southern blot and Western blot analysis. The complemented *csA* mutant (DLL5069) was named DLL5204 and the complemented *csB* mutant (DLL5071) was named DLL5208. All plasmid and genomic DNA isolation and manipulations were performed as outlined previously [61]. Nucleotide sequencing was performed by Micromon (Monash University, Melbourne, Australia) using a Prism BigDye terminator cycle sequencing kit according to the manufacturer’s instructions (Applied Biosystems, MA, USA).

### Confirmation of mutant and complemented strains by PCR and Southern hybridization

PCR was used to confirm the insertion of the targetron element in the appropriate genes or to confirm the loss of the targetron element upon complementation: for *csA*, oligonucleotide primers DLP111 (GCTATATAGAAACAAATGAAGTTGAAGATG) and DLP112 (CTCCATTTGATTTTTTACCATCAGTAA) were used; for *csB*, oligonucleotide primers DLP113 (AGTATTTACTGTAGATCCTAGACCTATGGG) and DLP114 (GGAATCTAGCTGTAAAAGGATCACAA) were used. A 1.8 kb size increase in the PCR product indicates the presence of the targetron element. For confirmation by Southern hybridization, purified genomic DNA was digested with either *Xba*I and *Ava*II (*csA*) or *Xba*I and *Pac*I (*csB*). Southern hybridization procedures were as outlined previously [61] and blots were hybridized either with an intron/*ermB*-specific probe [61], a *csA*-specific probe, generated using oligonucleotide primers DLP111 and DLP112, or a *csB*-specific probe, generated using oligonucleotide primers DLP113 and DLP114.

### Western blot analysis of CsA and CsB expression in mutant and complemented strains

To confirm the correct exosporial protein profiles of both mutants and complemented strains, exosporial proteins were chemically extracted from the spores of these strains [18] and the protein concentration determined using the BCA protein assay kit (Pierce) according to the manufacturer’s instructions. Western blot analysis was then performed as outlined previously [61] using 10 μg of protein from each strain which was separated by SDS-PAGE on 12% gels [67]. The Western blots were then probed using CsA- and CsB-specific antibodies. The antibodies were produced by Mimotopes (Melbourne, Australia) and generated by immunising rabbits with CsA and CsB specific peptides (CVFTDGKKSNGDDLDF and CEADDDENHNNHKCCK for CsA, CDRIFDFKCVNQQIPR and CLVVYSAPAEFKHHEK for CsB), with each peptide conjugated to a keyhole limpet hemocyanin. The extracted proteins were also visualised by separating 40 μg of protein on a 12% SDS-PAGE and staining with Coomassie Brilliant Blue G-250 (Sigma Aldrich).

### Immunogenic *C*. *sordellii* spore proteins

Western blot analysis was carried out as described for the Western blot analysis using CsA- and CsB-specific antibodies except that proteins were probed with antibodies raised against ATCC9714 whole spores [18]

### Expression and purification of recombinant CsA and CsB

To express CsA and CsB with a C-terminal 6xHis tag, *csA* was amplified with oligonucleotide primers DLP 143 (TATACCATGGGCATGAAGGATTTAATGAGAGC) and DLP 144 (CGTCTCGAGTTTACAACATTTGTGATTG) and CsB was amplified with oligonucleotide primers DLP147 (TATACCATGGGCATGACTAAAAATAATACAGC) and DLP148 (CGTCTCGAGTTTTTCGTGATGTTTAAATTC). The amplified DNA for CsA and CsB was digested with *Nco*I and *Xho*I and cloned into pET-28b (Novagen) to give rise to plasmid pDLL82 and plasmid pDLL83 for CsA and CsB, respectively. Plasmids pDLL82 and pDLL83 were individually introduced into strain BL21 (DE3)(C43) and the proteins were overexpressed at the Protein production Unit, Monash University, Australia, using Autoinduction Media (Overnight Express LB media, Merck). Protein purification was performed using nickel affinity chromatography and size exclusion chromatography (GE Superdex 200).

### Microscopy

Spores were prepared and visualized for TEM and SEM as previously described [18] All electron microscopy imaging was performed at the Monash CryoEM Ramaciotti Centre, Monash University, Australia. Whole spores that were imaged by TEM were analysed using FIJI software [68] to determine the inner spore and total spore lengths, inner spore width, position of the inner spore in relation to the exosporium and presence of an exosporium attached to the inner spore. For all strains, 10 spores per replicate were analysed from a total of four biological replicates. To determine differences between strains in the inner spore and total spore lengths and inner spore widths, the mean of the measurements for each biological replicate was calculated and this value was used for the statistical analysis. The positioning of the inner spore with respect to the exosporium was determined as follows: at each spore pole, the exosporium was measured as the length from the end of the inner spore at one pole to the end of the exosporium at the same pole. The pole with the longer exosporial length was then used to calculate the percentage of exosporium present at one pole using the formula (longer exosporial length at spore pole/total spore length) X 100. Spores were considered to have an inner spore centrally located within the exosporium if 35% or less of the exosporium was present at one pole. Spores were considered to have an inner spore positioned non-centrally within the exosporium if more than 35% of the exosporium was present at one pole.

### Sporulation assay

To compare the viable spores being produced by different strains, HIS broths were inoculated with overnight cultures of *C*. *sordellii* to a final OD_600_ of 0.1. The cultures were grown to an OD_600_ of 0.8 and then added to 20 ml TY broths at a 1 in 50 dilution. A sample of each culture was then taken every 24 hours for 72 hours. Half the sample was plated directly onto HIS agar to obtain total cell counts (spores and vegetative bacteria). The other half was heated at 65°C for 30 minutes to kill vegetative bacteria and then plated onto HIS agar to obtain viable spore counts alone. The ability of spores to form colonies was used as an indicator that viable spores had been produced during the sporulation assay.

### Spore resistance

To determine spore resistance, 1 x 10^6^ spores were treated with ethanol (80% v/v), hydrochloric acid (0.5 M), sodium hydroxide (0.15 M), lysozyme (1 mg/ml) or heat (75°C) for 30 minutes. With the exception of heat, all treatments were performed at 37°C. All spore stocks were in water with the exception of spores for lysozyme treatment where spores were in PBS. Percentage spore viability (the ability of spores to form colonies) following treatment was calculated as follows: (log_10_ CFU ml^-1^ after plating spores on HIS agar post treatment/log_10_ CFU ml^-1^ after plating untreated spores on HIS agar) X 100.

### Spore colony forming efficiency assay

The concentration of spores in all samples was standardized to approximately 6 x 10^7^ spores ml^-1^ by counting the total numbers of spores present using a hemocytometer (spores able to and spores unable to form colonies). The viable spore count for each sample was then determined by plating spores onto HIS agar and obtaining the CFU ml^-1^. Total spore counts and viable spore counts were compared to determine any differences in colony forming efficiencies between spores of the wild-type and mutant strains.

### Adherence of spores to vaginal and cervical cell lines

Spores were tested for their ability to adhere to or be internalized by the following cell lines: VK2/E6E7 vaginal epithelial cell line (ATCC CRL-2616), End1/E6E7 endocervical epithelial cell line (ATCC CRL-2615) and Ect1/E6E7 ectocervical epithelial cell line (ATCC CRL-2614). Cell lines were cultured as previously described [69], seeded at 2.5 x 10^5^ cells ml^-1^ in 24-well culture plates and grown to 95% confluency. The cells were washed with PBS and 200 μl (2.5 x 10^6^ cells ml^-1^) of *C*. *sordellii* viable spores, resuspended in antibiotic free media, added to each well at an multiplicity of infection of 10:1 and incubated at 37°C in an atmosphere of 5% CO_2_. Samples were then processed as described previously [30] to obtain the percentage of bound spores, with the exception that cells were lysed in 0.1% triton X-100 in a final volume of 400 μl and samples plated onto HIS agar. Briefly, following incubation, infected cells were washed to remove unbound spores and the cells were lysed to obtain the number of adhered and/or internalized spores. This was compared to infected cells which were directly lysed to obtain the total spore numbers. The percentage of adhered and/or internalized spores was calculated as follows: (CFU ml^-1^ adhered and/or internalized spores/ CFU ml^-1^ total spores) X 100.

### Ethics statement

Animal handling and experimentation was performed according to the requirements of the 7^th^ edition of the *Australian Code of Practice for the care and use of animals for scientific purposes* (2004) and The Victorian Prevention of Cruelty to Animals Act (1986), and was approved by the Monash Animal Research Platform Committee under license number MARP/2014/145.

### Mouse gastrointestinal tract model of *C*. *sordellii* infection

Groups of five pathogen-free six to eight week old wild type male C57BL/six mice (Walter and Eliza Hall Institute of Medical Research) were treated with antibiotics for seven days followed by three days of normal drinking water, as previously described [70] with the exception that gentamicin was administered at 0.07 mg/ml. Mice were then infected with 10^7^ viable *C*. *sordellii* spores by oral gavage and monitored for disease symptoms of diarrhea, weight loss and behavioral changes. A minimum of five mice were orally gavaged with each strain. Mice were humanely euthanized at the completion of the experiment. Faecal samples were collected daily to monitor for *C*. *sordelii* spore shedding. Faecal samples were then resuspended in PBS at a final weight per volume ratio of 100 mg ml^-1^, heated at 65°C for 30 minutes to kill vegetative cells and then plated onto HIS agar containing d-cycloserine (250 μg/ml), kanamycin (20 μg/ml), streptomycin (20 μg/ml), trimethoprim (20 μg/ml) and naladixic acid (20 μg/ml). A Tukey post-hoc test was performed to determine significant weight loss differences between mice and variability in spore shedding between mice infected with each strain.

### Growth kinetics

Starter cultures of *C*. *sordellii* strains were grown overnight in HIS broths. The broths were then diluted to an OD_600_ of 0.02 in fresh HIS broth and a 200 μl volume was added to wells in a 96-well tray (Grenier Bio-One). Trays were incubated in an anaerobic chamber and removed each hour for a period of nine hours to measure the absorbance on a Tecan plate reader at 600 nm. A Kruskal-Wallis test was performed to determine differences in growth between the strains.

### Statistical analysis

GraphPad Prism was used for all statistical analysis. A Mann-Whitney test was performed with a 95% confidence interval unless otherwise stated in which case a Kruskal-Wallis test was performed for the statistical analysis of the *C*. *sordellii* growth kinetics or a Tukey post-hoc test was performed for the statistical analysis of the weight loss between mice and the variability in spore shedding between mice infected with each strain in the mouse gastrointestinal tract model of *C*. *sordellii* infection.

## Supporting information

S1 Fig Wild-type spores after chemical treatment to remove the exosporium. Whole spores imaged by transmission electron microscopy. Scale bar = 0.5 μm.
(TIF)

S2 Fig Analysis of *C*. *sordellii* exosporial proteins. Recombinant CsA and recombinant CsB probed with anti-CsA (A) and anti-CsB (B) antibodies, respectively. Wild-type exosporial extracts probed with anti-CsB antibodies showing the presence of two prominent protein bands (C). Exosporial extracts visualised on a Coomassie stained SDS-PAGE (D). Wild-type and *csA* mutant exosporial extracts probed with anti-whole spore antibodies (E). Expected molecular weights of recombinant CsA, recombinant CsB, CsA and CsB are 46 kDa, 44 kDa, 45 kDa and 43 kDa, respectively. Wild-type, WT; recombinant CsA, rCsA; recombinant CsB, rCsB; *csA* mutant, *csA*-M; *csA* mutant complemented, *csA*-C; *csB* mutant, *csB*-M; *csB* mutant complemented, *csB*-C.
(TIF)

S3 Fig Confirmation of mutants and complemented strains by Southern hybridization. Genomic DNA was digested with *Xba*I/*Ava*II for Southern hybridization (A) and for DNA extracted from the wild-type, *csA* mutant and *csA* complemented strains for Southern hybridization (C). Genomic DNA was digested with *Xba*I/*Pac*I for Southern hybridization (B) and for DNA extracted from the *csB* mutant and *csB* complemented strains for Southern hybridization (C). Hybridization using *csA* (A) and *csB* (B) specific probes showed an increase in fragment size of 1.8 kb in the *csA* and *csB* mutants corresponding to the insertion of the targetron element. Hybridization with an intron specific probe (C) also showed fragments of the correct size for the *csA* and *csB* mutants. The targetron vector pJIR3566 (V) was used as a control for the intron specific probe. Wild-type, WT; *csA* mutant, *csA*-M; *csA* mutant complemented, *csA*-C; *csB* mutant, *csB*-M; *csB* mutant complemented, *csB*-C.
(TIF)

S4 Fig Morphological features and dimensions of *C*. *sordellii* mutant and wild-type spores. Wild-type (A), *csA* (B) and *csB* (C) mutant spore sections imaged by transmission electron microscopy. Spores of the *csA* mutant have an inner spore that is positioned towards one pole of the exosporium. Thus the percentage of the exosporium present at one spore pole is recorded here (D) with each point representing the value of an individual spore. Each symbol represents one biological sample (circle, square, arrowhead, diamond). Solid horizontal lines indicate the median value. Dotted horizontal lines indicate the value above which a spore was considered to have an inner spore positioned toward one pole of the exosporium (exosporial length at one pole was greater than 35% of total spore length). A detached exosporium in the *csB* mutant spore sample, as imaged by whole mount transmission electron microscopy (TEM) (E). The mean inner spore length (F) and width (G) and total spore length (H) of wild-type and mutants strains with measurements performed on whole spores imaged by TEM. The total spore length includes the length of the inner spore and the exosporium. The results represent the average of four independent biological experiments and error bars represent standard error of the mean. Wild-type, WT; *csA* mutant, *csA*-M; *csA* mutant complemented, *csA*-C; *csB* mutant, *csB*-M; Ex, exosporium; IS, inner spore. Scale bar: images A-C = 1 μm, image E = 0.5 μm.
(TIF)

S5 Fig Spore colony forming efficiency of untreated spores and resistance of *csA* and *csB* mutant spores to lysozyme and ethanol. To determine the ability of mutant versus wild-type spores to form colonies, spores were grown on sporulation media, purified and counted either with a haemocytometer to determine the total number of spores present, which includes the viable spores (able to form colonies) and non-viable spores (unable to form colonies) or plated onto HIS agar to obtain only the number of viable spores (A). To determine spore resistance, spores were incubated in the presence of 1 mg/ml lysozyme (B) or 80% ethanol (v/v) (C) for 30 minutes after which the spores were plated on HIS agar to determine the number of viable spores remaining following treatment. The results represent the average of at least four independent biological experiments and error bars represent standard error of the mean. Wild-type, WT; *csA* mutant, *csA*-M; *csB* mutant, *csB*-M.
(TIF)

S6 Fig Growth curves of *C*. *sordellii* wild-type and mutant strains. Growth in HIS broths were monitored over a nine-hour period with the OD_600_ recorded every hour. Wild-type (red circle), *csA* mutant (green square), *csB* mutant (blue arrowhead). The results represent the average of four independent biological experiments and error bars represent standard error of the mean. Wild-type, WT; *csA* mutant, *csA*-M; *csB* mutant, *csB*-M.
(TIF)

S1 Table Raw proteomics data for proteins identified by LC MS/MS in the exosporial extracts of *C*. *sordellii* ATCC9714. Protein and peptide summaries are presented for exosporial extracts of two independent biological spore stocks.
(XLSX)

S2 Table Proteins identified by LC MS/MS in the exosporial extracts of *C*. *sordellii* strain ATCC9714. *C*. *difficile* exosporial proteins [29] that showed homology to *C*. *sordellii* proteins are recorded here as gene identifications. Only proteins that were identified with at least one unique peptide in both biological replicates have been included in this table.
(PDF)

S1 Appendix Gene and protein sequences of CsA and CsB. (PDF)
