# Supplementary figures and images for: Clostridium sordellii outer spore proteins maintain spore structural integrity and promote bacterial clearance from the gastrointestinal tract
Source: PLoS Pathog. 2018 Apr 18;14(4):e1007004. doi: 10.1371/journal.ppat.1007004 (PMC5927469; doi:10.1371/journal.ppat.1007004)

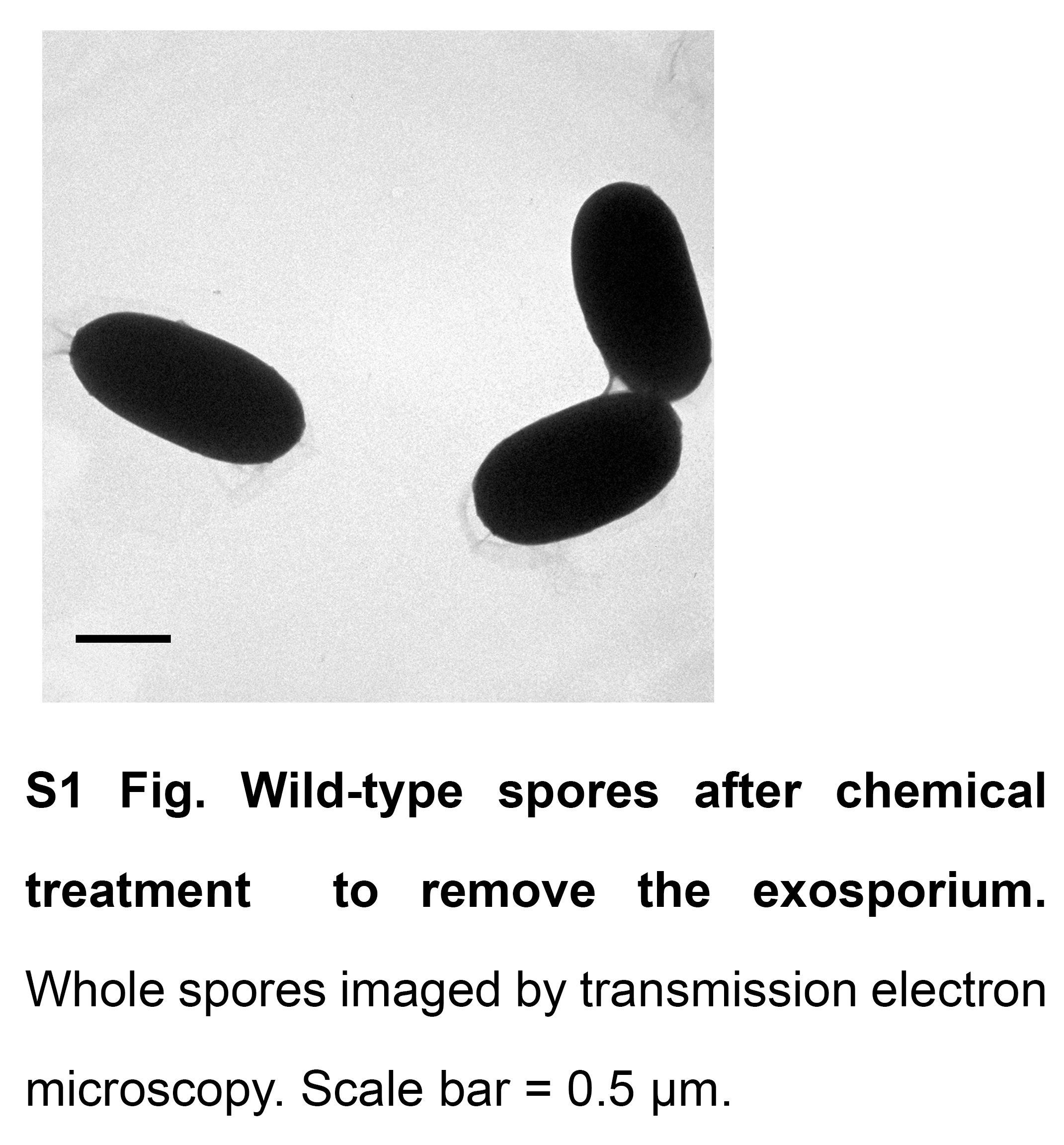

Supplement: S1 Fig — Whole spores imaged by transmission electron microscopy. Scale bar = 0.5 μm. (TIF) [file ppat.1007004.s001.tif]

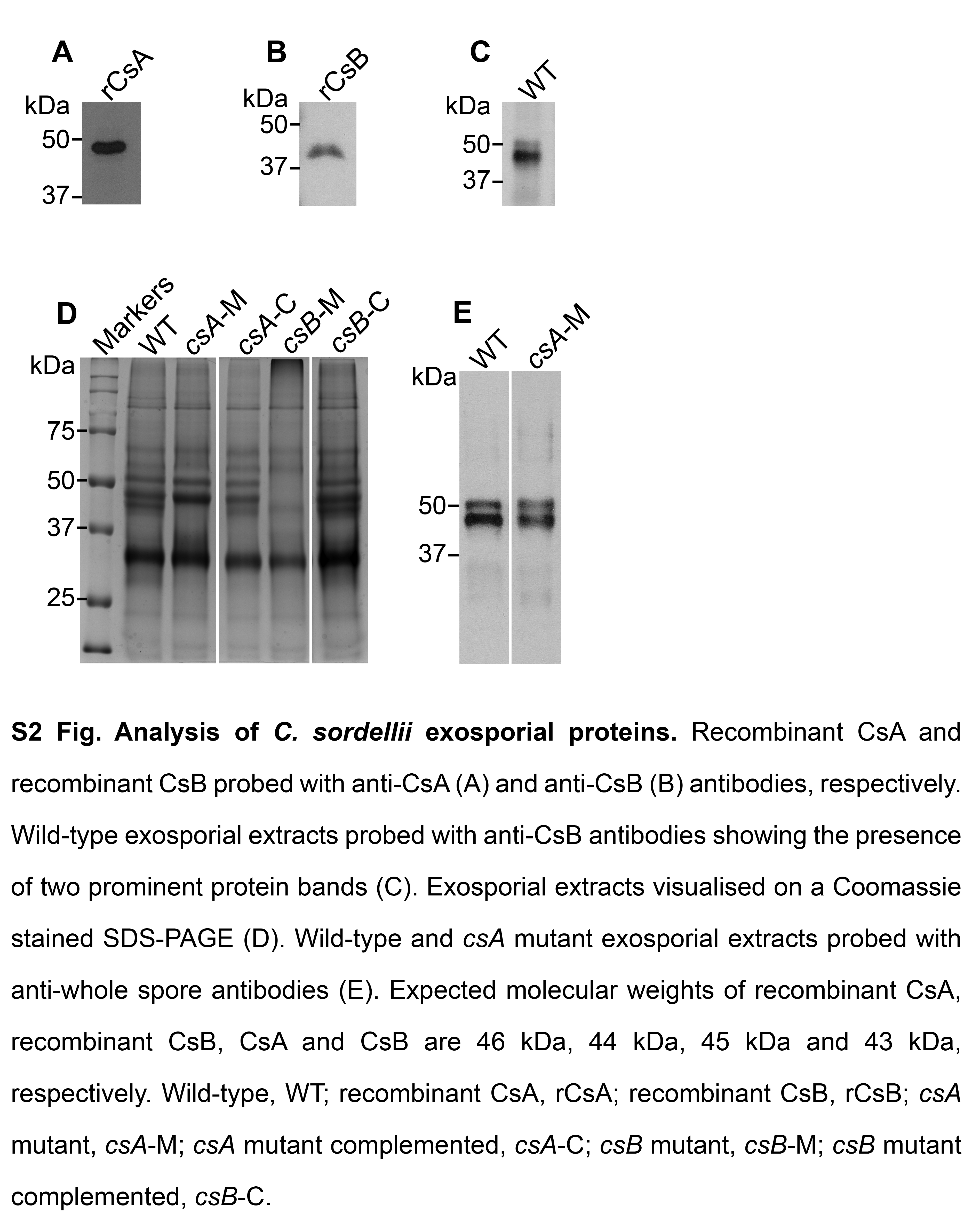

Supplement: S2 Fig — Recombinant CsA and recombinant CsB probed with anti-CsA (A) and anti-CsB (B) antibodies, respectively. Wild-type exosporial extracts probed with anti-CsB antibodies showing the presence of two prominent protein bands (C). Exosporial extracts visualised on a Coomassie stained SDS-PAGE (D). Wild-type and csA mutant exosporial extracts probed with anti-whole spore antibodies (E). Expected molecular weights of recombinant CsA, recombinant CsB, CsA and CsB are 46 kDa, 44 kDa, 45 kDa and 43 kDa, respectively. Wild-type, WT; recombinant CsA, rCsA; recombinant CsB, rCsB; csA mutant, csA-M; csA mutant complemented, csA-C; csB mutant, csB-M; csB mutant complemented, csB-C. (TIF) [file ppat.1007004.s002.tif]

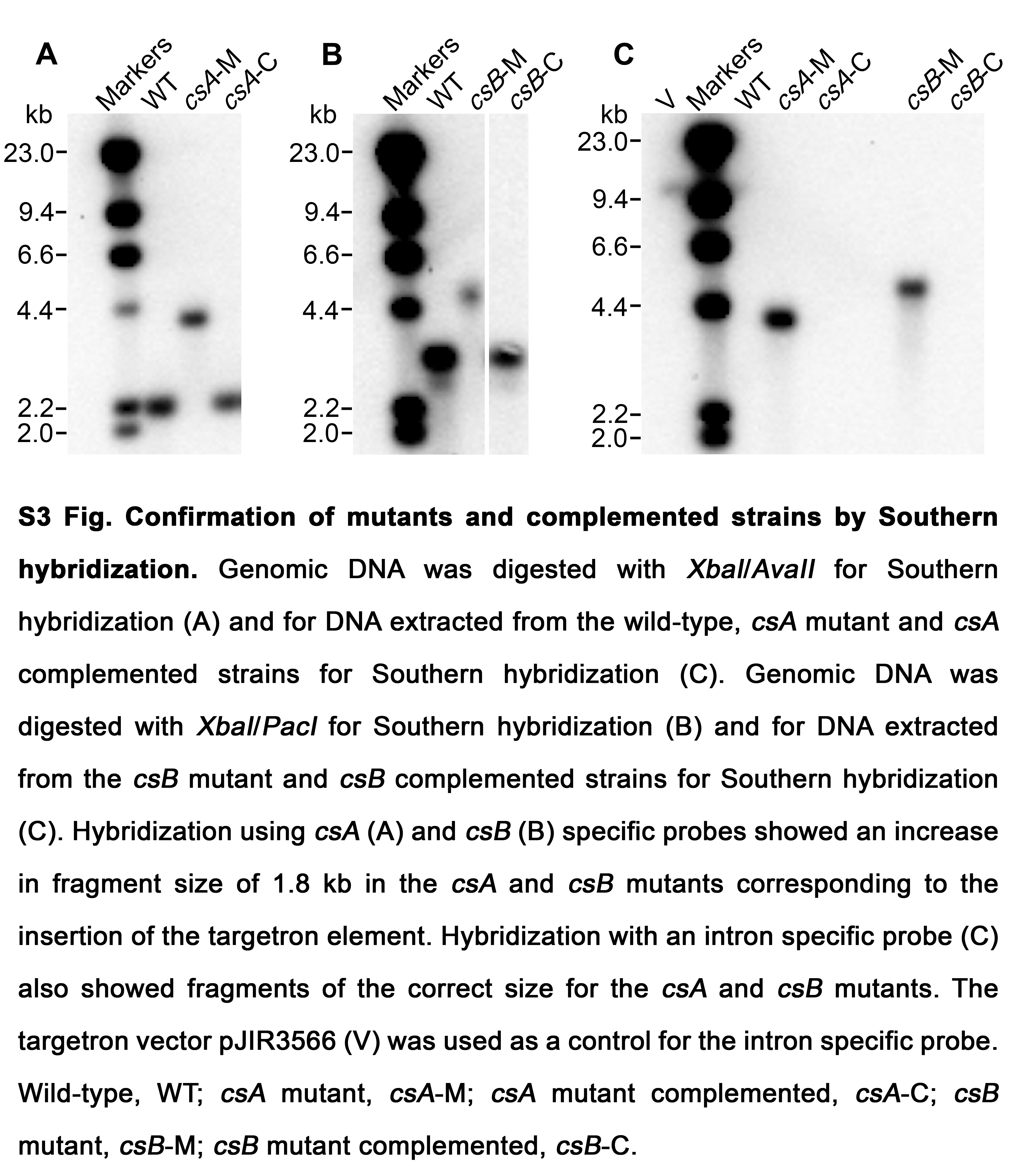

Supplement: S3 Fig — Genomic DNA was digested with XbaI/AvaII for Southern hybridization (A) and for DNA extracted from the wild-type, csA mutant and csA complemented strains for Southern hybridization (C). Genomic DNA was digested with XbaI/PacI for Southern hybridization (B) and for DNA extracted from the csB mutant and csB complemented strains for Southern hybridization (C). Hybridization using csA (A) and csB (B) specific probes showed an increase in fragment size of 1.8 kb in the csA and csB mutants corresponding to the insertion of the targetron element. Hybridization with an intron specific probe (C) also showed fragments of the correct size for the csA and csB mutants. The targetron vector pJIR3566 (V) was used as a control for the intron specific probe. Wild-type, WT; csA mutant, csA-M; csA mutant complemented, csA-C; csB mutant, csB-M; csB mutant complemented, csB-C. (TIF) [file ppat.1007004.s003.tif]

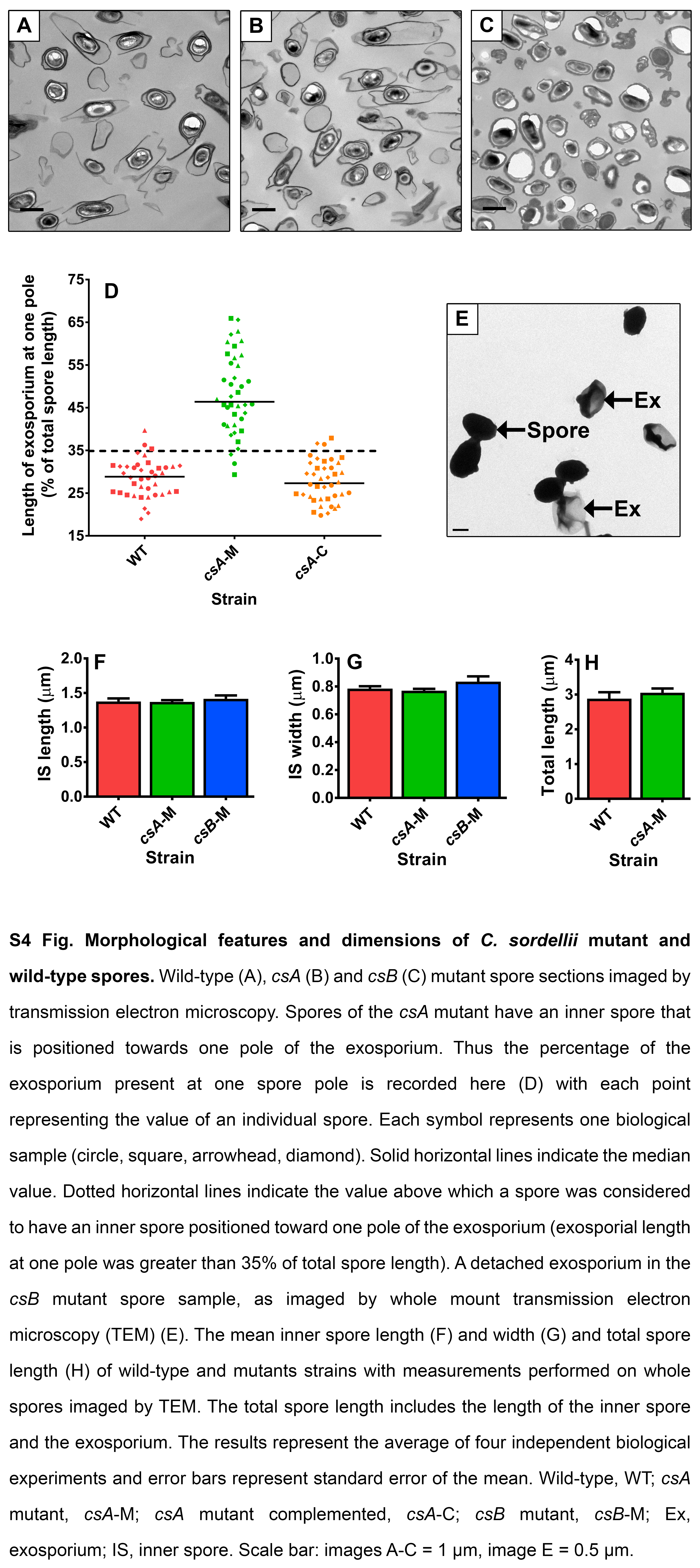

Supplement: S4 Fig — Wild-type (A), csA (B) and csB (C) mutant spore sections imaged by transmission electron microscopy. Spores of the csA mutant have an inner spore that is positioned towards one pole of the exosporium. Thus the percentage of the exosporium present at one spore pole is recorded here (D) with each point representing the value of an individual spore. Each symbol represents one biological sample (circle, square, arrowhead, diamond). Solid horizontal lines indicate the median value. Dotted horizontal lines indicate the value above which a spore was considered to have an inner spore positioned toward one pole of the exosporium (exosporial length at one pole was greater than 35% of total spore length). A detached exosporium in the csB mutant spore sample, as imaged by whole mount transmission electron microscopy (TEM) (E). The mean inner spore length (F) and width (G) and total spore length (H) of wild-type and mutants strains with measurements performed on whole spores imaged by TEM. The total spore length includes the length of the inner spore and the exosporium. The results represent the average of four independent biological experiments and error bars represent standard error of the mean. Wild-type, WT; csA mutant, csA-M; csA mutant complemented, csA-C; csB mutant, csB-M; Ex, exosporium; IS, inner spore. Scale bar: images A-C = 1 μm, image E = 0.5 μm. (TIF) [file ppat.1007004.s004.tif]

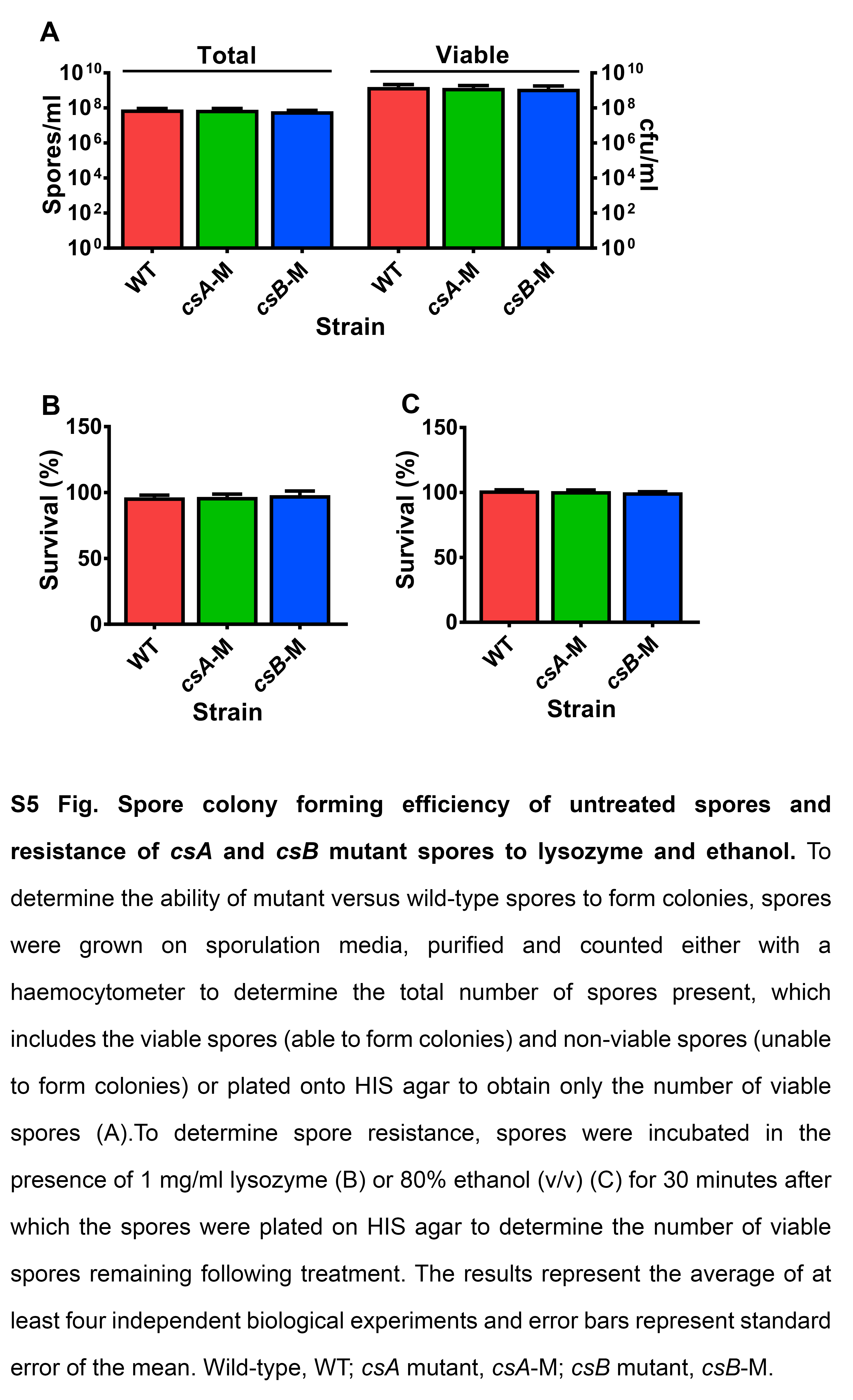

Supplement: S5 Fig — To determine the ability of mutant versus wild-type spores to form colonies, spores were grown on sporulation media, purified and counted either with a haemocytometer to determine the total number of spores present, which includes the viable spores (able to form colonies) and non-viable spores (unable to form colonies) or plated onto HIS agar to obtain only the number of viable spores (A). To determine spore resistance, spores were incubated in the presence of 1 mg/ml lysozyme (B) or 80% ethanol (v/v) (C) for 30 minutes after which the spores were plated on HIS agar to determine the number of viable spores remaining following treatment. The results represent the average of at least four independent biological experiments and error bars represent standard error of the mean. Wild-type, WT; csA mutant, csA-M; csB mutant, csB-M. (TIF) [file ppat.1007004.s005.tif]

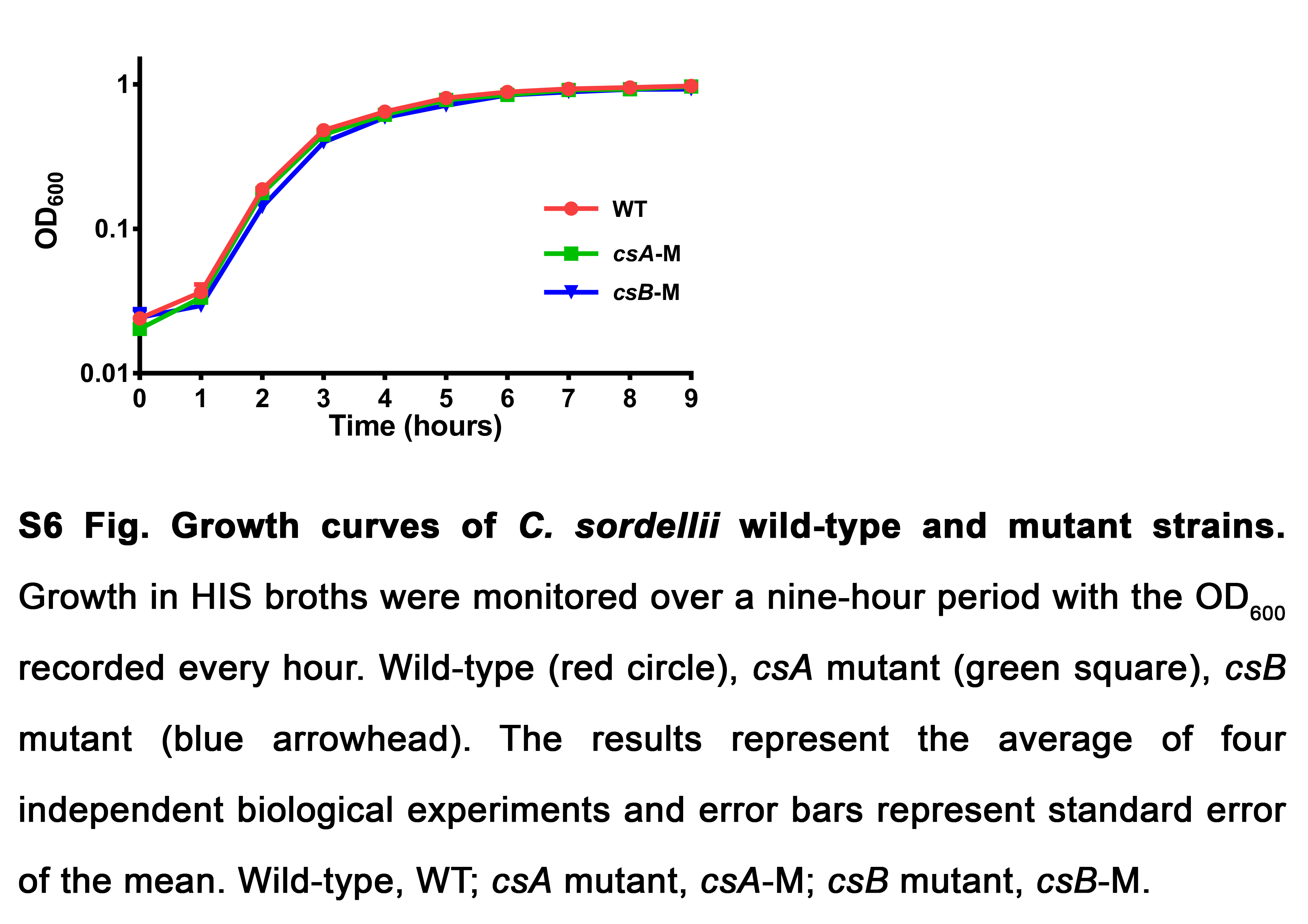

Supplement: S6 Fig — Growth in HIS broths were monitored over a nine-hour period with the OD600 recorded every hour. Wild-type (red circle), csA mutant (green square), csB mutant (blue arrowhead). The results represent the average of four independent biological experiments and error bars represent standard error of the mean. Wild-type, WT; csA mutant, csA-M; csB mutant, csB-M. (TIF) [file ppat.1007004.s006.tif]
