# Supplementary material for: Clostridium sordellii outer spore proteins maintain spore structural integrity and promote bacterial clearance from the gastrointestinal tract
Source: PLoS Pathog. 2018 Apr 18;14(4):e1007004. doi: 10.1371/journal.ppat.1007004 (PMC5927469; doi:10.1371/journal.ppat.1007004)
Supplement: S2 Table — C. difficile exosporial proteins [29] that showed homology to C. sordellii proteins are recorded here as gene identifications. Only proteins that were identified with at least one unique peptide in both biological replicates have been included in this table. (PDF) [file ppat.1007004.s008.pdf]

**S2 Table**

| Protein description | Gene identification | UniProt ID | Number peptides [a] | *C. difficile* |
|---|---|---|---|---|
| **Germination** | | | | |
| Peptidase M20/M25/M40 family protein | H477_0435 | T0E6L8 | 8/7 | - |
| Peptidase M20/M25/M40 family protein | H477_0436 | T0CTM7 | 3/2 | - |
| Putative peptidoglycan binding domain protein | H477_0372 | T0D2M7 | 3/7 | - |
| **Pathogenicity** | | | | |
| Translation elongation factor Tu | H477_0026 (*tuf*) | T0D4X3 | 15/23 | CD0071 (*tuf1*) |
| TCP-1/cpn60 chaperonin family protein | H477_0241 | T0D415 | 4/4 | CD0194 (*groL*) |
| Hsp70 family protein | H477_2996 | T0CNF5 | 7/5 | CD2461 (*dnaK*) |
| Phosphopyruvate hydratase | H477_4609 (*eno*) | T2REI3 | 1/4 | CD3170 (*eno*) |
| **Spore assembly** | | | | |
| Glycosyl hydrolases 18 family protein | H477_2042 | T0E6S9 | 10/10 | CD1433 (*cotE*) |
| Manganese containing catalase family protein | H477_3485 | T0DWI1 | 4/2 | CD2401 (*cotD*) |
| Manganese containing catalase family protein | H477_3486 | T0DR76 | 3/4 | - |
| Uncharacterized protein | H477_3144 | T0DXD7 | 52/21 | CD1067 (*cdeC*) |
| **Spore resistance** | | | | |
| Reverse rubrerythrin-1 | H477_0313 | T0D302 | 4/4 | - |
| Reverse rubrerythrin-1 | H477_0314 | T0EAX3 | 4/4 | - |
| Uncharacterized protein | H477_2973 | T0DRL7 | 7/12 | CD1511 (*cotB*) |
| Small, acid-soluble spore protein beta | H477_4660 | T2RDK8 | 7/16 | - |
| **Unknown putative role** | | | | |
| Putative amidase domain protein | H477_1207 | T0E528 | 1/1 | - |
| Uncharacterized protein | H477_1422 | T0E0I6 | 16/7 | - |
| Uncharacterized protein | H477_1423 | T0CMC7 | 12/5 | - |
| Coat F domain protein | H477_1527 | T0E0R1 | 1/1 | - |
| Uncharacterized protein | H477_1731 | T0E5Y2 | 1/3 | - |
| Cupin domain protein | H477_1872 | T0E1S4 | 1/1 | - |
| Uncharacterized protein | H477_1876 | T0E6D8 | 2/3 | |
| Uncharacterized protein | H477_2974 | T0CDB2 | 1/5 | - |
| Uncharacterized protein | H477_3325 | T0CLP4 | 6/5 | - |
| Uncharacterized protein | H477_3327 | T0DW21 | 9/5 | - |
| Uncharacterized protein | H477_3692 | T2RFS0 | 1/4 | - |
| Uncharacterized protein | H477_3966 | T2RGS3 | 3/2 | |
| Uncharacterized protein | H477_4099 | T2RG98 | 23/29 | - |
| Uncharacterized protein | H477_4384 | T2RFG7 | 3/8 | - |
| Fascin domain protein | H477_5266 | T0EB21 | 2/3 | - |
| Uncharacterized protein | H477_5615 | T0D6Z2 | 1/1 | - |

| | | | | |
|---|---|---|---|---|
| **Cytosolic proteins** | | | | |
| 50S ribosomal protein L7/L12 | H477_0034 (*rplL*) | T0CV21 | 4/3 | NA |
| Small GTP-binding domain protein | H477_0047 | T0ECN6 | 1/2 | NA |
| 30S ribosomal protein S8 | H477_0067 (*rpsH*) | T0D495 | 1/3 | NA |
| S4 domain protein | H477_0085 | T0E826 | 2/1 | NA |
| 50S ribosomal protein L17 | H477_0088 (*rplQ*) | T0D4U1 | 2/2 | NA |
| Peptidyl-prolyl cis-trans isomerase | H477_0324 | T0EAY0 | 2/2 | NA |
| Acyl-CoA dehydrogenase, N-terminal domain protein | H477_0355 | T0E6E7 | 4/2 | NA |
| CO dehydrogenase/acetyl-CoA synthase complex beta subunit | H477_0479 | T0EB97 | 3/1 | NA |
| Pyruvate formate lyase family protein | H477_0509 | T0D3J0 | 3/2 | NA |
| Branched-chain-amino-acid aminotransferase | H477_0714 (*ilvE*) | T0CZT3 | 2/1 | NA |
| Glycine/sarcosine/betaine reductase complex component C subunit beta domain protein | H477_0807 | T0E3Z3 | 1/1 | NA |
| Acetate kinase (Acetokinase) | H477_1003 (*ackA*) | T0CRP9 | 1/1 | NA |
| Propanediol utilization PduL family protein | H477_1156 | T0E9N3 | 1/1 | NA |
| Pyruvate formate lyase family protein | H477_1816 | T0E668 | 2/2 | NA |
| Peroxiredoxin | H477_2044 | T0CP16 | 9/8 | NA |
| Putative membrane protein | H477_2616 | T0CFK2 | 2/3 | NA |
| Elongation factor Ts | H477_2726 (*tsf*) | T0CFX2 | 1/2 | NA |
| 30S ribosomal protein S2 | H477_2728 (*rpsB*) | T0CQV5 | 3/2 | NA |
| M42 glutamyl aminopeptidase family protein | H477_2794 | T0DZF4 | 1/1 | NA |
| Tryptophanase 2 | H477_3251 (*tnaA2*) | T0CM84 | 3/2 | NA |
| ATP synthase subunit beta, sodium ion specific | H477_4865 (*atpD*) | T2RDU1 | 3/6 | NA |
| ATP synthase F1, alpha subunit | H477_4869 | T2RCL9 | 1/2 | NA |
| M42 glutamyl aminopeptidase family protein | H477_4964 | T2RCT6 | 2/4 | NA |

[a] Number of unique peptides in replicate 1/ number of unique peptides in replicate 2.

**S2 Table. Proteins identified by LC MS/MS in the exosporial extracts of *C. sordellii* strain ATCC9714.** *C. difficile* exosporial proteins [1] that showed homology to *C. sordellii* proteins are recorded here as gene identifications. Only proteins that were identified with at least one unique peptide in both biological replicates have been included in this table.

**Reference**

1. Díaz-González F, Milano M, Olguin-Araneda V, Pizarro-Cerda J, Castro-Córdova P, Tzeng SC, et al. Protein composition of the outermost exosporium-like layer of *Clostridium difficile* 630 spores. Journal of Proteomics. 2015;123:1-13. doi: 10.1016/j.jprot.2015.03.035.
