# Supplementary material for: Clostridium sordellii outer spore proteins maintain spore structural integrity and promote bacterial clearance from the gastrointestinal tract
Source: PLoS Pathog. 2018 Apr 18;14(4):e1007004. doi: 10.1371/journal.ppat.1007004 (PMC5927469; doi:10.1371/journal.ppat.1007004)
Supplement: S1 Appendix — (PDF) [file ppat.1007004.s009.pdf]

S1 Appendix. Gene and protein sequences of CsA and CsB

CsA protein sequence

MKDLMRAKKVVSKRVTKPTQELENSSYIETNEVEDDFERGCKSSGHKPQCHPYVQCECEC
ECDCDCNHEHNHKHNHDCHSSCCNCNEDDCTENCFPNPCPEECSQPLTPPKFPISQAVLY
PIETNRVFDTIMFRVFTDGKKSNGDDLDFKYELGNLCGPLPSSARTNVTIEKVCINYSSI
KINPGTTSLEQLKIEKVDRKSQKCKSVFEYDVCGQLNRCCNDKCLGQNVAYKEKGLNVVV
YDLELELTGRFGCSEFTAVAIPCSKCESKHDNSNCVVFYFNTLSGDLVVPADGRGFTLRQ
DFQTQLTVDCIGKGVITQKMVNNKPCYELDIPNGIDLVLCLQNVVSALIEEQIIVLGGPT
RIRPRLVDTFGSVCDFSQFTGKCEADDDENHNNHKCCK

csA gene sequence

ATGAAGGATTTAATGAGAGCTAAAAAAGTAGTTAGCAAAAGAGTAACAAAGCCTACTCAA
GAGTTAGAAAATAGTAGCTATATAGAAACAAATGAAGTTGAAGATGATTTTGAAAGAGGA
TGCAAATCTAGTGGACACAAACCCCAATGTCATCCATATGTACAATGCGAGTGTGAATGT
GAGTGTGATTGCGATTGTAATCATGAACACAATCATAAGCATAACCATGATTGTCACTCA
AGTTGCTGTAATTGCAATGAAGATGATTGTACAGAGAATTGCTTCCCAAACCCTTGTCCA
GAAGAGTGTAGTCAGCCTTTAACACCACCTAAGTTTCCTATATCACAAGCAGTTCTATAC
CCTATAGAAACAAATAGAGTATTTGATACTATAATGTTTAGAGTATTTACTGATGGTAAA
AAATCAAATGGAGATGATTTAGACTTTAAATATGAATTAGGTAACTTATGTGGACCATTA
CCAAGTTCTGCTCGTACAAATGTAACTATAGAAAAAGTATGCATAAACTACTCAAGTATA
AAAATTAACCCAGGGACAACATCATTAGAGCAATTAAAGATTGAAAAAGTTGATAGAAAA
TCACAAAAATGTAAGTCAGTTTTTGAATATGATGTTTGTGGTCAACTAAATAGATGCTGT
AATGATAAGTGCTTAGGGCAAAATGTAGCATACAAAGAAAAAGGTTTAAATGTAGTTGTA
TATGACTTAGAACTAGAATTAACAGGTAGATTTGGATGTTCAGAGTTTACTGCTGTAGCG
ATACCATGTAGTAAATGTGAATCAAAACATGACAATTCAAACTGTGTAGTATTCTATTTT
AATACTTTATCTGGTGATTTAGTAGTTCCAGCAGATGGAAGAGGATTTACTCTAAGACAA
GACTTCCAGACTCAATTAACAGTTGACTGTATAGGAAAAGGTGTAATAACTCAAAAAATG
GTTAACAATAAACCATGCTATGAGTTAGATATACCAAATGGAATAGATCTTGTACTTTGT
TTACAAAATGTAGTAAGTGCATTAATAGAAGAACAAATAATAGTTTTAGGTGGACCAACA
AGAATTAGACCTAGATTAGTAGACACATTTGGTAGTGTTTGTGACTTCTCTCAATTCACA
GGAAAATGTGAAGCTGACGATGATGAAAATCATAACAATCACAAATGTTGTAAATAA

CsB protein sequence

MTKNNTAQTNSNIDNLTCDRLNSNQYRESQECNCYPERPSYDHCPERPQYGHCYEKPQYD
HCPEKPQYGHCHEKPQYDHCPEKPQYNHCHEKPQHDYCPERPQYDPCKGNKKPNCCCCSE
AKFNQNECGKPCNPVKFECPDINCAICTPILADRIFDFKCVNQQIPRSIAPQVFTVDPRP
MGCPPYTAGAPVCIEKVTVVYDCLGFSNDLLPITLCNTSGSLTGTPQLCPSGEASVFHNY
EGTISTGCICREKGSTATLVQGPLDASIYNFQYIVEGFIGCDPFTARFPVTPSDAPDFLT

NINLFSTICLPNTDDPLTLRAQFDLKFVVKSIVPTTPFSGGTFSARIFDELLISEKLYLT
KSDALVVYSAPAEFKHHEK

csB gene sequence

ATGACTAAAAATAATACAGCTCAAACAAACAGTAATATCGATAATCTTACTTGTGATAGA
TTAAACTCTAATCAATATAGAGAAAGTCAAGAATGTAATTGTTATCCTGAAAGACCTAGC
TATGATCATTGTCCAGAAAGACCTCAATACGGCCATTGTTATGAGAAACCTCAATATGAT
CATTGCCCTGAGAAACCTCAGTATGGTCATTGTCATGAAAAGCCTCAGTATGATCATTGC
CCTGAGAAACCTCAATACAATCATTGTCATGAAAAACCTCAACATGATTATTGCCCTGAA
AGACCTCAGTATGACCCATGTAAAGGTAATAAAAAACCAAATTGCTGTTGTTGTTCAGAA
GCAAAATTTAATCAAAATGAATGTGGTAAACCTTGTAACCCTGTGAAATTTGAATGCCCA
GATATAAACTGTGCTATATGTACACCTATATTGGCAGATAGAATATTTGATTTCAAATGT
GTAAATCAACAAATACCTAGATCTATAGCACCTCAAGTATTTACTGTAGATCCTAGACCT
ATGGGATGTCCTCCATATACAGCTGGAGCTCCTGTATGTATAGAAAAAGTTACTGTAGTA
TACGATTGTTTAGGGTTCTCAAATGATTTATTACCTATAACTTTATGTAATACTAGCGGT
TCACTTACAGGAACACCTCAACTATGTCCTTCAGGTGAAGCATCTGTATTCCATAATTAT
GAAGGAACAATATCTACAGGTTGTATTTGTCGTGAGAAAGGTTCAACTGCAACTTTAGTT
CAAGGACCTTTGGATGCATCTATTTATAACTTCCAATATATAGTTGAAGGATTTATAGGT
TGTGATCCTTTTACAGCTAGATTCCCAGTAACTCCATCAGATGCTCCTGATTTCTTAACA
AATATAAACTTATTTAGCACTATTTGTTTACCTAATACTGATGATCCATTAACTTTAAGA
GCTCAATTTGATCTTAAATTCGTAGTTAAATCTATAGTTCCAACAACACCTTTCAGCGGA
GGAACTTTCAGTGCTAGAATATTTGATGAATTATTAATTAGTGAAAAACTATACCTTACA
AAATCAGATGCACTAGTTGTATACTCAGCTCCTGCTGAATTTAAACATCACGAAAAATAA
